# Intentional release of native species undermines ecological stability

**DOI:** 10.1073/pnas.2218044120

**Published:** 2023-02-07

**Authors:** Akira Terui, Hirokazu Urabe, Masayuki Senzaki, Bungo Nishizawa

**Affiliations:** ^a^Department of Biology, University of North Carolina at Greensboro, Greensboro, NC 27412; ^b^Salmon and Freshwater Fisheries Research Institute, Hokkaido Research Organization, Eniwa, Hokkaido 061-1433, Japan; ^c^Faculty of Environmental Earth Science, Hokkaido University, Sapporo, Hokkaido 060-0810, Japan; ^d^National Institute of Polar Research, Tachikawa, Tokyo 190-8518, Japan

**Keywords:** coexistence theory, competition, resilience, ecological modeling, fishery

## Abstract

The massive release of captive-bred native species (“intentional release”) is a pervasive method to enhance wild populations of commercial and recreational species. However, such external inputs may disrupt the sensitive species interactions that allow competing species to coexist, potentially compromising long-term community stability. Here, we use theory and long-term data of stream fish communities to show that intentional release destabilizes community dynamics with limited demographic benefit to the enhanced species. Our theory predicted that intentional release intensifies interspecific competition, facilitating the competitive exclusion of unenhanced species that otherwise stably coexist. In parallel, the excessive input of captive-bred individuals suppressed the natural recruitment of the enhanced species via intensified within-species competition. Consequently, the ecological community with the intentional release is predicted to show reduced community density with unstable temporal dynamics. Consistent with this prediction, stream fish communities showed greater temporal fluctuations and fewer taxonomic richness in rivers with the intensive release of hatchery salmon—a major fishery resource worldwide. Our findings alarm that the current overreliance on intentional release may accelerate global biodiversity loss with undesired consequences for the provisioning of ecosystem services.

## Significance

The intentional release of captive-bred individuals is a common practice for conservation and natural resource management. However, we know little about its potential consequences for the whole ecological community. Here, we show that the intentional release undermines community stability with limited demographic benefit to the enhanced species. Theory and data agree that intentional release destabilizes community dynamics by facilitating competitive exclusion while suppressing the natural recruitment of the enhanced species. The effect size of the intentional release was striking in its magnitude, doubling temporal fluctuations of enhanced communities compared to those with no intentional release. Our findings point to major limitations of intentional release as a primary tool for conservation and sustainability.

Human demands for natural resources are ever-increasing, such that active interventions are critical to the sustainable management of fisheries, forestry, and wildlife (1). Releases of captive-bred native species (“intentional release”) is a form of the efforts to enhance wild populations of diverse taxa (1, 2). In particular, the magnitude of intentional release for commercial and recreational species is massive (1, 3)—large-scale programs release thousands to millions of plant (4), invertebrate (crustaceans, bivalves, insects) (3, 5, 6), and vertebrate individuals (fish, birds) (3, 7) into the wild annually. There is growing awareness that intentional release entails ecological risks, such as the accumulation of deleterious alleles and the intensified competition within the enhanced species (8–10). Yet, this method is still pervasive in natural resource management owing to the significant economic benefit (3, 11–13). For example, Kitada (3) analyzed the economic performance of release programs for 12 major fishery species across the globe. The gross monetary yield exceeded the seed-production cost in nine species, suggesting that release programs are economically profitable in general.

The intentional release, however, can have wider-ranging consequences than previously thought, as the impacts can propagate through a diversity of ecological interactions (14). This recognition has sparked a discussion of how the massive introduction of native species alters short-term ecosystem dynamics (15–17). Yet, current debates overlook the fact that we have rarely assessed the community-wide impact in the long term. Ecological theory suggests that the stable coexistence of competing species through density-dependent feedback underpins the emergent stability of ecological communities (18–20). Intentional release may disrupt the sensitive balance of species interactions because it introduces unnaturally high numbers of individuals into the wild (1, 3, 14). Hence, this form of species management may intervene in the ecological process that allows competing species to coexist, ultimately degrading the long-term community stability. Evidence for this hypothesis is lacking, however.

Here, we show that intentional release undermines long-term community stability, which we define as the relative size of fluctuations in total community density over time (18). Our theory illuminates that intentional release compromises the stabilizing mechanism emerging from species niche differences. The present study further demonstrates the relevance of this general theory to natural systems by showing its congruence with Japanese stream fish communities, where ~10 million hatchery masu salmon (*Oncorhynchus masou masou*) are released annually for fisheries, recreation, and conservation purposes across the nation (21). Once released as fry, masu salmon stay in freshwater for at least 1 y before sea migration and compete for resources with other stream-dwelling species. Therefore, this system serves as an excellent model for studying the community-wide impact of intentional release. Our integrative approach provides strong evidence for the destabilizing effect of intentional release on ecological communities.

## Results and Discussion

### Theoretical Prediction.

We employed a multi-species Ricker model (22) to simulate community dynamics with the intentional release of a constituent species (species 1). Specifically, the population density of species i at time t+1, $N_{i,t+1}$, is modeled as follows:

$$\begin{array}{c} N_{i,t+1}=(N_{i,t}+\varphi_{i}R_{t})\\ \;exp[r_{i}(1-\frac{\alpha_{i1}R_{t}+\sum_{j=1}^{S}\alpha_{\mathrm{ij}}N_{j,t}}{K_{i}})]exp\left(\varepsilon_{i,t}\right), \end{array}$$

where $r_{i}$ is the intrinsic growth rate, $\alpha_{\mathrm{ij}}$ the competition coefficient of species j on species i ($\alpha_{\mathrm{ii}}=1$), $K_{i}$ the carrying capacity, $R_{t}$ the number of released individuals for the enhancement of species 1, and $\varepsilon_{i,t}$ the species response to stochastic environmental fluctuations that follow a normal distribution $\mathrm{Normal}(0,\sigma_{\varepsilon }^{2})$. The parameter $\varphi_{i}$ controls the relative fitness of captive-bred individuals as follows:

$$\varphi_{i}=\{\begin{array}{cc} f_{R} & \left(i=1\right)\\ 0 & \left(i\neq 1\right) \end{array},$$

$f_{R}\left(\geq ,0\right)$ is the density-independent survival of captive-bred individuals relative to wild individuals. Therefore, the model accounts for the fitness difference of captive-bred individuals due to genetic effects and/or plasticity (2, 23, 24) when considering the reproductive contribution to the next generation. Without loss of generality, we assumed constant $K_{i}$ ($K_{i}=K$) and $R_{t}$ ($R_{t}=R$) across species and time, respectively.

Prior to our full-community analysis, we analyzed a two-species community to understand the model behavior. The prior analysis revealed distinct parameter spaces predicting destabilizing, neutral, and stabilizing effects of intentional release on community dynamics (*SI Appendix*, Figs. S1 and S2). In general, the intentional release had a neutral to destabilizing effect in a community with slow-growing species ($r_{i}<1.5$). Meanwhile, for higher values of $r_{i}$ (≥1.5), intentional release stabilized community dynamics by dampening limit cycles or chaos. Carrying capacity and competition also played an important role in shifting the parameter spaces. According to this result, we chose 32 simulation scenarios, differing in the intrinsic growth of an enhanced species $r_{1}$, average competition strength $\bar{\alpha }$, carrying capacity K, and relative fitness $f_{R}$ (*Methods*). To capture variation in species traits, intrinsic growth rates of unenhanced species and interspecific competition coefficients were drawn randomly from a uniform ($r_{i,i\neq 1}∼\mathrm{Unif}(0.5,2.5)$) and an exponential distribution ($\alpha_{ij,i\neq j}∼\mathrm{Exp}(1/\bar{\alpha })$). Under each parameter scenario, we ran 1,500 time steps of 1,000 independent communities (i.e., simulation replicates). Using the last 1,000 time steps, we obtained the following summary statistics of the total community density $\sum_{i}^{S}N_{i}$ to examine the community-level response to intentional release: coefficient of variation (CV), number of species persist (defined as $N_{i}>0.01$ for the entire simulation), temporal mean (μ), and temporal SD (σ). We also calculated the temporal mean and SD for the enhanced ($N_{1}$) and unenhanced species ($\sum_{i,i\neq 1}^{S}N_{i}$) separately to infer underlying mechanisms.

**Fig. 1.**
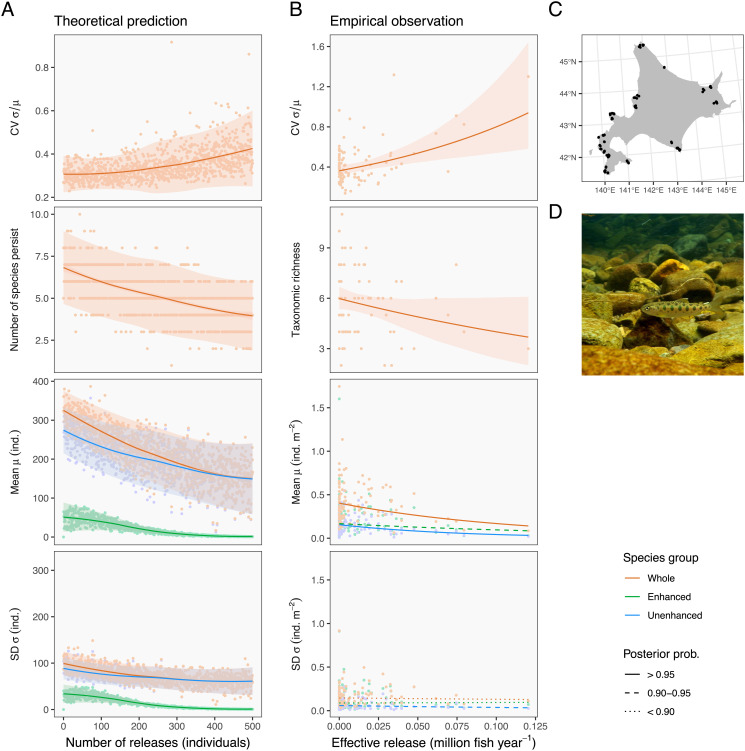
Theory and empirical observations agree that intentional release destabilizes community dynamics. In *A* and *B*, panels and colors distinguish response variables and species groups. (*A*) Theoretical predictions. Dots represent individual simulation replicates, and lines are the loess curves fitted to simulated data with their 95% confidence (dark shade) and prediction intervals (light shade). Parameters used in this simulation are intrinsic growth rate of an enhanced species $r_{1}=$ 1.5 average interspecific competition $\bar{\alpha }=$ 0.25, carrying capacity K= 100, environmental stochasticity $\sigma_{\varepsilon }=$ 0.5, and relative fitness of captive-bred individuals $f_{R}=$ 1. These parameter values are comparable to those estimated in the empirical Ricker model. (*B*) Empirical evidence. Dots represent observations at 97 sites within 31 watersheds. Lines and shades are the predicted values and their 95% credible intervals of the regression models, and line types correspond to the coefficient’s posterior probabilities. Effective release is a latent explanatory variable estimated in our hierarchical Bayesian model. See *Methods* for details. Full statistics were reported in *SI Appendix*, Tables S6–S8. (*C*) Map of sampling sites (black dots) in Hokkaido, Japan. (*D*) Masu salmon *Oncorhynchus masou masou*. Photo credit: Akira Terui.

When small carrying capacity was combined with a low to modest intrinsic growth rate (K=100, $r_{1}\leq 2.5$), our model predicted a destabilizing effect of intentional release on ecological communities, as illustrated by increased CV with increasing numbers of releases (Fig. 1*A*). This pattern stemmed mainly from the reduced mean of the total community density, and both enhanced and unenhanced species groups were responsible (Fig. 1*A*). The enhanced species decreased because release induced the negative competitive effect exceeding the reproductive contribution of released individuals, as reported in a previous theoretical study (25). Meanwhile, interspecific competition reduced the unenhanced species, resulting in fewer persisting species (Fig. 1*A*). Combined, the total community density decreased more sharply than individual species groups (Fig. 1*A*). The SDs showed a similar trend, but the relationship was flatter at the community level (Fig. 1*A*). Since a CV is a ratio of an SD to a mean, the steeper decline of the mean community density led to the increased CV. These patterns were qualitatively similar across ecological contexts with the low carrying capacity (*SI Appendix*, Figs. S3–S6), but except for the scenario in which the enhanced species exhibits chaos ($r_{1}=$ 3.5).

The destabilizing effect emerges because intentional release affects the balance of species interactions that underpins community stability. In theory, stable coexistence requires a niche difference that is large enough to overcome the relative difference in intrinsic competitive ability (19). Under this condition, competing species can grow from small populations because dominant species undergo stronger intraspecific competition (19). Such coexistence favors stable temporal dynamics of species-rich communities (18) because it gives rise to “overyielding” (19, 20), i.e., total community density of a multi-species community exceeds what would be expected in a single-species community ($\sum_{i}^{S}N_{i}>K$). However, the intentional release is externally controlled, and the number of releases is not subject to density-dependent regulation. Therefore, released individuals impose additional intra- and interspecific competition that interferes with the ecological process producing overyielding.

The intentional release had little or a stabilizing influence on community dynamics when carrying capacity was sufficiently large (K= 400; *SI Appendix*, Figs. S7–S10). In particular, intentional release increased the enhanced species with a low population growth rate ($r_{1}=$ 0.5; *SI Appendix*, Fig. S7), suggesting that weak intraspecific competition within the enhanced species (=$r_{1}K^{-1}$) is needed for intentional release to be effective. The weak-competition scenario is likely in the reintroduction of extirpated species or long-lived endangered species. In fact, some of the best evidence for successful intentional release comes from conservation programs of such species (26–29). In the Sado Island (Japan), for example, the once-extirpated Crested Ibis *Nipponia nippon* showed exponential growth of the population since the initial reintroduction of 10 captive-bred individuals in 2008 (28). Similarly, translocations of Humpback chub, a federally listed fish species in the United States, seem highly cost-effective in enhancing their tributary populations in the Little Colorado River (29). However, the preponderance of unsuccessful reintroduction in conservation implies that the theoretical requirements for successful intentional release are not always met (6, 30, 31). Indeed, non-negligible numbers of projects seem to release captive-bred individuals into unsuitable habitats with compromised carrying capacities (6, 30). Our analysis suggests that such programs could rather impair biodiversity without noticeable demographic benefit to the enhanced species. Pre-release examination and restoration of environmental capacity may be key to successful release programs with minimal impacts on other community members.

### Empirical Evidence.

To demonstrate the relevance of our general theory to natural systems, we assessed the potential impacts of the intentional release of masu salmon (Fig. 1*D*) on the long-term stability of stream fish communities in Hokkaido, Japan. In the protected watersheds (all separated by the ocean; Fig. 1*C*), a long-term program exists to monitor stream fish communities along with the official release records. The release of masu salmon began in the 1950s; the release numbers vary due mainly to logistical reasons, while several of the watersheds are not subject to intentional release to preserve regional populations. This resulted in the average annual release ranging from 0 (no release) to 0.24 million fish across watersheds during 1999 to 2019. Most release occurs in spring near fish survey locations, after which salmon fry stays in freshwater for at least 1 y with other stream fishes (see *SI Appendix*, Fig. S16 for a co-occurrence matrix in our dataset). Therefore, the study system sets the stage for a “natural experiment” to test our theoretical predictions. We used the data from 1999 to 2019 at 97 sites within 31 independent watersheds (see *Methods* for selection criteria). Using hierarchical Bayesian models, we quantified the effect of intentional release on community dynamics while accounting for the potential effects of climates and local abiotic factors.

**Fig. 2.**
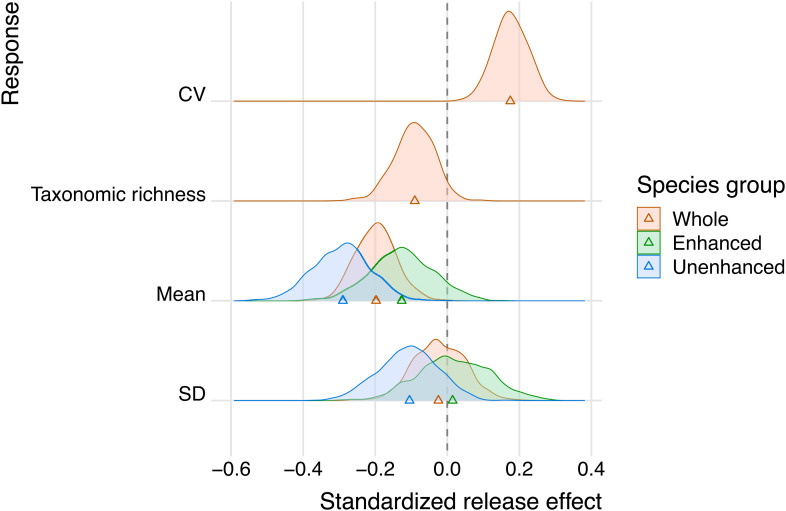
Posterior distributions for the standardized regression coefficients of intentional release. Y-axis represents different response variables grouped by colors distinguishing species groups (whole community, enhanced species [masu salmon], and unenhanced species). Triangles indicate median estimates.

As predicted, stream fish communities showed greater temporal fluctuations (higher CV) at sites with the intensive release (Fig. 1*B*). The effect was striking in its magnitude, almost doubling the CV at the highest release level. Our analysis strongly supported the positive relationship between the CV and the number of “effective” releases (Fig. 2; see *Methods* for the Bayesian estimation of effective release), in which the probability of the regression coefficient being positive was 1.00 (*SI Appendix*, Table S6). This pattern was associated with the reduced long-term average of the total community density and fewer taxonomic richness (Figs. 1*B* and 2), and both enhanced (masu salmon) and unenhanced fish groups contributed to this trend (Figs. 1*B* and 2; see *SI Appendix*, Tables S6–S8 for full statistics). In the meantime, the SDs had vague relationships with the intentional release (Figs. 1*B* and 2).

**Fig. 3.**
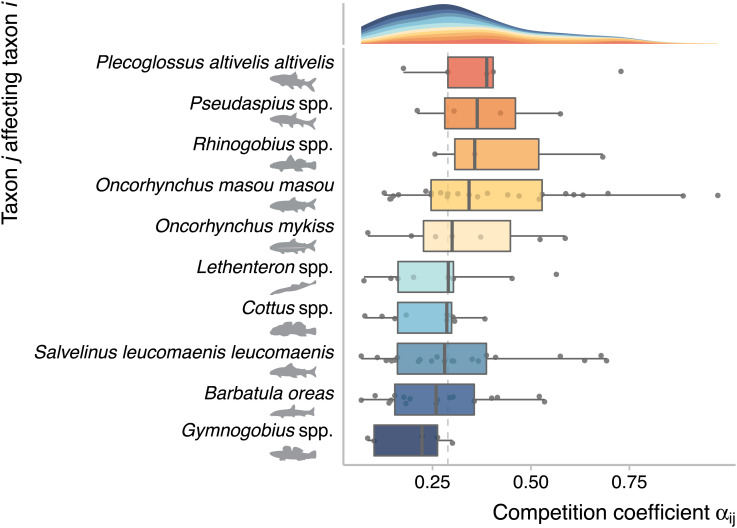
Estimated competition coefficients of the multi-species Ricker model. Dots represent a given pair of taxa, and box plots show median (vertical center line) and quartiles (box limits) with whiskers extending up to the most extreme data points that are within ±1.5 interquartile range. The density plot on the top shows the overall distribution of the competition coefficient. Colors distinguish taxa. The vertical broken line denotes the overall median value.

To gain better insights into the driving mechanisms, we fitted a multi-species Ricker model to a subset of our time-series data (nine sites with few missing observations and no intentional release; *Methods*). The parameter estimates of this model are directly comparable to those used in the theoretical Ricker model. The estimates of interspecific competition coefficients $\alpha_{\mathrm{ij}}$ ranged 0.07 to 0.97 with the median of 0.29 (Fig. 3), suggesting that competition is a likely mechanism as our theory assumes (see *SI Appendix*, Fig. S17 for all pairwise estimates). Our statistical inference is supported by previous studies. For example, competition between masu salmon and other salmonid species is well documented, and its form can be interference or exploitative (32–34). Diets of salmonid species are innately overlapped, and they interfere with each other to claim profitable foraging spots for drift insects (33) or cruise over a large area in search of prey (35). Exploitation competition between masu salmon and benthic fish (e.g., sculpins and stone loach in our study system) is also likely because subordinate masu salmon in the dominance hierarchy consumes benthic macroinvertebrates (36) that many benthic fish species prey on (37, 38). Importantly, hatchery masu salmon may intensify intra- and interspecific competition in the wild (39–41). Hatchery salmon are larger and more aggressive than wild individuals of a comparable life stage, increasing the likelihood of intense intra- and interspecific competition (23). Indeed, field and experimental studies confirmed that hatchery masu salmon competed with wild masu salmon and other stream fishes during their freshwater life stage (39–41).

The reduced fitness of hatchery masu salmon may also play a role in the observed response. Like other salmonids, masu salmon exhibit a life history of “partial migration”; in our study region, the majority of individuals migrate to the ocean and spend approximately 1 y before spawning, although some males stay in freshwater for their entire life (42–44). It has been shown that ocean-migrating adults of hatchery masu salmon show lower return rates to the spawning river (45), and such genetic effects may accumulate during successive generations in captivity (8). Typically, masu salmon are reared in captivity for two to four generations. In light of the 70-y period of the extensive release program, it is conceivable that the “hatchery gene” has spread within the wild populations with measurable fitness declines. This effect may add to negative density dependence to influence community stability.

External control of intentional release is a theoretical premise for the destabilizing effect to emerge, and this is exactly how the release of masu salmon is operated. The hatchery program aims to produce and release a constant number of hatchery masu salmon every year, although uncontrollable factors (e.g., disease, budget allocation) generate some variation in the release number between years. Therefore, the number of releases is determined without accounting for the current condition of the recipient ecosystem. As such, released fish are probably “excessive” and may cause resource competition that would otherwise not exist.

It is noteworthy that the scale of the release program at the protected watersheds (annual average maximum = 0.24 million fish) is comparable or even smaller when compared with those for other fishery resources (1, 3). For example, 649.1 million fish are released annually to supplement the pink salmon stock at the Prince William Sound in Alaska, where wild populations seem to be severely compromised due to competition with hatchery fish (46). Similarly, the large-scale release of black sea bream has been conducted in Kagoshima Bay (Japan) since 1974 (a yearly average of 0.64 million juveniles) with associated declines of wild stocks and genetic diversity (47). Although we do not know the carrying capacities of the recipient systems, it is clear that our results should not be viewed as an exceptional case. In support of this view, a meta-analysis revealed that only a few massive release programs in fisheries led to an increased abundance of the focal species (24). Yet, none of those studies statistically quantified the community-level impact. Future studies addressing this knowledge gap are therefore desired to generalize our findings.

Two unique strengths in our dataset may have helped uncover the qualitative agreement between theory and empirical patterns. First, human activities are strictly regulated in the protected watersheds (*SI Appendix*, Fig. S14). Any form of exploitation, including angling, is prohibited in these areas. In addition, exclusive permission from the governor of Hokkaido is required to physically alter in-stream and riparian habitats. Therefore, unmeasured human influences should have minimal influences on the observed relationships. As such, we were able to confine potential confounding factors to natural environmental variation, which was properly accounted for in our statistical model (*Methods*). Second, the sampling method in this monitoring program (a combination of cast net and electrofishing) is carefully chosen to capture stream fishes with different microhabitat preferences (e.g., water-column vs. bottom). Thus, our data are robust to potential sampling bias. Nevertheless, our results must be viewed with some caution: We cannot exclude the possibility of spurious correlations due to the inherent nature of correlative analysis and field research. Since an experimental approach is nearly impossible at this spatial scale, the application of novel causal inferences (e.g., convergent cross-mapping) (48–50) may be a potential alternative to confirm the causal relationship between intentional release and community dynamics. Unfortunately, these methods were not applicable to our data because they require a longer and non-sparse time series to yield robust results.

### Implications.

Despite the significant attention to the fate of captive-bred individuals (2, 24), current schemes rarely consider the self-regulation process of biodiversity. Our results suggest that ignorance of this critical process may erode the long-term persistence of the recipient community, likely impacting the stable delivery of ecosystem services (51). We anticipate that the detrimental community-level consequence is not rare, or even pervasive, because many release programs are designed to aid declining populations (6, 30, 31, 52). In rural Spain, two million individuals of reared red-legged partridge, the most important game bird in this area, are introduced into the wild every year to overcome the regional decline of this species (7). In the United States, a nationwide initiative exists to augment populations of freshwater mussels, which have rapidly disappeared over the past decades for enigmatic reasons (6). The exact causes behind such population declines are often unknown or controversial, yet it is reasonable to assume that those habitats are no longer suitable and can support limited numbers of individuals (i.e., low carrying capacity). As long as this important theoretical condition of limited carrying capacity holds true, the phenomenon observed in our study streams may occur broadly across taxa and ecosystems.

While socioeconomic analysis is required to provide detailed guidance on release programs, it is clear that habitat conservation should be prioritized for the sustainability of natural resources. Protected areas and environmental restoration are promising tools to conserve biodiversity, and a smart spatial design is integral to achieving successful conservation. For example, coordinated placement of conservation sites considering spatial biodiversity patterns is crucial in improving the ecological outcomes (53–56). Governance may also play a central role in enforcing environmental legislation, potentially determining the effectiveness of conservation investment (57). These considerable potentials indicate that viable management options exist before blindly accepting intentional release. Without a comprehensive framework that appreciates the ecological integrity of natural communities, the intentional release will never be effective but impairs biodiversity.

## Methods

### Theory.

#### Model.

We employed a multi-species Ricker model (22). In the basic formula without intentional release, the population density of species i at time t+1, $N_{i,t+1}$, is modeled as follows:

[1] $$N_{i,t+1}=N_{i,t}exp[r_{i}(1-\frac{\sum_{j=1}^{S}\alpha_{\mathrm{ij}}N_{j,t}}{K_{i}})]exp\left(\varepsilon_{i,t}\right),$$

where $r_{i}$ is the intrinsic growth rate, $\alpha_{\mathrm{ij}}$ the competition coefficient of species j on i, $K_{i}$ the carrying capacity, and $\varepsilon_{i,t}$ the species response to stochastic environmental fluctuations that obey a normal distribution $\mathrm{Normal}(0,\sigma_{\varepsilon }^{2})$. We modified this formula to include the effects of intentional release (species 1) on reproduction and competition as follows:

[2] $$N_{i,t+1}=(N_{i,t}+\varphi_{i}R_{t})exp[r_{i}\{1-\frac{\alpha_{i1}(N_{1,t}+R_{t})+\sum_{j=2}^{S}\alpha_{\mathrm{ij}}N_{j,t}}{K_{i}}\}]exp\left(\varepsilon_{i,t}\right),$$

$R_{t}$ is the number of released individuals, and the parameter $\varphi_{i}$ controls the relative fitness of captive-bred individuals:

[3] $$\begin{array}{c} \varphi_{i}=\{\begin{array}{cc} f_{R} & \left(i=1\right)\\ 0 & \left(i\neq 1\right) \end{array} \end{array},$$

$f_{R}\left(\geq ,0\right)$ is the density-independent survival of captive-bred individuals relative to wild individuals. Eq. **2** can be reorganized to:

[4] $$\begin{array}{c} N_{i,t+1}=(N_{i,t}+\varphi_{i}R_{t})exp[r_{i}(1-\frac{\alpha_{i1}R_{t}+\sum_{j=1}^{S}\alpha_{\mathrm{ij}}N_{j,t}}{K_{i}})]exp\left(\varepsilon_{i,t}\right) \end{array}.$$

In this model, intrinsic growth rates of unenhanced species $r_{i,i\neq 1}$ and interspecific competition $\alpha_{\mathrm{ij}}$ are random draws from a uniform ($r_{i,i\neq 1}∼\mathrm{Unif}(0.5,r_{max})$) and an exponential distribution ($\alpha_{ij,i\neq j}∼\mathrm{Exp}(1/\bar{\alpha })$), respectively. We assumed constant values of intraspecific competition ($\alpha_{\mathrm{ii}}=1$), carrying capacity $\left(K_{i}=K\right)$ and the number of releases $\left(R_{t}=R\right)$.

### Model Analysis.

First, we analyzed a two-species community to gain insights into the model behavior and to guide the N-species analysis. Specifically, we examined the release effect with 20 parameter values of intrinsic population growth rate $r_{i}$ (0.5 to 3.5 with an equal interval) and carrying capacity K (50 to 500 with an equal interval), resulting in 400 parameter combinations. In a single-species Ricker model, the chosen range of $r_{i}$ can generate stable equilibrium, damped oscillations, limit cycle, and chaos (58). For simplicity, we assumed $r_{1}=r_{2}$. We crossed this parameter setup with environmental stochasticity ($\sigma_{\varepsilon }=$ 0, 0.5) and competition strength ($\alpha_{\mathrm{ij}}=$ 0.25, 0.5) and generated 1,600 simulation scenarios (400×2×2).

Under each simulation scenario, we ran 1,500 timesteps of community dynamics for 100 values of release level R (0 to 500 with an equal interval). We introduced 50 individuals of each species and allowed them to grow with no intentional release for the first 100 timesteps (initialization). After the initialization period, we released R individuals of the enhanced species every timestep over the next 400 timesteps to reach a new equilibrium with the intentional release (burn-in period). We continued the simulation run with intentional release and saved the last 1,000 timesteps, which were used to obtain the following summary statistics of the whole community $\sum_{i}^{S}N_{i}$ (S: the number of species): the CV, the temporal mean (μ), and the SD (σ). The results of the two-species system were reported in *SI Appendix, Text*.

We subsequently analyzed a whole community model with 10 species using important parameter combinations identified in the two-species simulation: intrinsic growth of enhanced species ($r_{1}=$ 0.5, 1.5, 2.5, 3.5), average strength of interspecific competition ($\bar{\alpha }=$ 0.25, 0.5), and carrying capacity (K= 100, 400). We also varied the relative fitness of captive-bred individuals ($f_{R}=$ 0.5, 1) as this is a common interest in captive-breeding programs. Meanwhile, we fixed values of the following parameters: the number of species (S= 10), maximum intrinsic growth rate of unenhanced species ($r_{\max}=$ 2.5), and environmental stochasticity ($\sigma_{\varepsilon }=$ 0.5). We used $r_{\max}=$ 2.5 because the intrinsic growth rate higher than this value was rarely observed in nature (59). This simulation setup resulted in 32 sets of parameter combinations.

As with the two-species model, we ran 1,500 timesteps of community dynamics with 1,000 values of release level R (0 to 500 with a constant interval) for each simulation scenario. In this simulation, we initialized the community by introducing five individuals of each species and repeated this seeding with Poisson draws every 10 timesteps to account for possible complex dynamics of a 10-species community. In addition, we defined a threshold density ($N_{i}=0.01$) below which species are removed from the simulation (i.e., an absorbing condition) and are recorded as “extinct.” The rest of the simulation procedure is identical to the two-species model. We summarized the values of simulation parameters in *SI Appendix*, Table S1.

### Empirical analysis.

#### Data.

##### Time-series data.

We assembled time-series fish data at 126 sites within 32 protected watersheds of Hokkaido Island, Japan. The Hokkaido Research Organization leads a long-term monitoring program at these watersheds, and the data are published as annual reports (60). The program began in 1963, but an effective, standardized sampling method has been implemented since 1999; at all sampling sites, a combination of electrofishing and cast net (two-pass) was used to effectively catch both benthic and water-column species (*SI Appendix, Text*). Most data were collected in summer with irregular interannual intervals (1- to 3-y intervals for most cases), and sampling efforts were quantified by sampling area (average: 175.49 ± 115.91 m^2^). We confined our analysis to the sites that meet the following criteria: i) The observation span (from the first to the last year of observation) exceeds 10 y, ii) the number of observation years exceeds 5 y, and iii) masu salmon is observed at least twice during the observation period. As a result, we used time-series data at 97 sites within 31 watersheds from 1999 to 2019. The summed abundance of first and second passes was used in the following analysis. *SI Appendix*, Table S2 summarizes observed species in these watersheds.

##### Fish release.

The release of masu salmon began in the 1950s. The duration of captivity ranged from zero (wild origin) to seven generations, and the majority is released at two to four generations in captivity. Although the program aims to release a constant number of hatchery fish for each watershed, uncontrollable factors (e.g., disease, budget allocation) produce some variation in the number of releases among years. Hatchery fish are released in spring (fry and smolt stages) and fall (juvenile stage). Most fish are released near the fish survey sites. However, the number of fish released was reported as an aggregate for each watershed, and the site-specific information of release is unavailable. For each release stage, we assembled annual records of intentional release (the number of fish released; 1999 to 2019) from annual investigations by the Japan Fisheries Research and Education Agency and Salmon and Freshwater Fisheries Research Institute. During the study period, the majority of release took place in spring at a fry stage (fry: juvenile: smolt = 1: 0.09: 0.41)

##### Environmental data.

At each sampling site, we measured the following environmental variables as potential covariates: upstream watershed area (km^2^; a proxy for stream size), proportional land use in the upstream watershed (forest, urban, agriculture), local climates (annual mean air temperature [C°] and cumulative precipitation [mm]), and ocean productivity (sea surface chlorophyll *a* concentration [mg m^−3^]). We used MERIT Hydro (61) to delineate the upstream watershed polygon for each sampling site. We estimated the proportion of forest, urban, and agriculture in each watershed polygon based on land use data in 2015 from Copernicus Global Land Service (100-m resolution) (62). Climate data at each sampling site were extracted from CHELSA version 1.2 (63, 64). We extracted annual data of chlorophyll *a* concentration (2002 to 2019; resolution, 4.6 km^2^) from OceanColor (65) as a proxy for ocean productivity and calculated the average value within the 30-km radius of each river mouth. We used the following R packages to perform the geospatial analysis: *sf* (66), *terra* (67), *exactextractr* (68), *stars* (69), and *whitebox* (70)*.*

### Statistical Analysis.

#### Community dynamics comparison.

Our goal is to compare temporal community dynamics across sites. However, the data are not comparable because of observation errors (e.g., different observers) and missing observations. To confront this challenge, we developed a Bayesian state-space model for two species groups separately: i) enhanced species, the abundance of masu salmon, and ii) unenhanced species, the summed abundance of all species except masu salmon. A Bayesian state-space model is best suited for our analysis because it can account for observation errors while imputing missing values given the long-term trend at each site (71, 72). The model is composed of observation and state models, as described below.

In the observation model, we model observation processes. Fish abundance at site s in year t (either enhanced or unenhanced species), $N_{s,t}$, was assumed to follow a Poisson distribution:

[5] $$\begin{array}{c} N_{s,t}∼\mathrm{Poisson}(\lambda_{s,t}A_{s,t}) \end{array},$$

where $\lambda_{s,t}$ is the expected fish density (individual m^-2^) and $A_{s,t}$ the sampling area (m^2^). Since fish sampling was conducted after the spring release of masu salmon, captured fish may include individuals released in the observation year. We explicitly modeled this observation process to avoid biases in estimating temporal community trends:

[6] $$\begin{array}{c} \lambda_{s,t}=n_{s,t}exp\left(\varepsilon_{s,t}^{\mathrm{obs}}\right)+\psi \beta_{s}Fry_{w\left(s\right),t} \end{array},$$

$n_{s,t}$ is the “true” fish density excluding fish released in the spring, $Fry_{w\left(s\right),t}$ the number of salmon fry released (unit: million fish) in spring in watershed w within which site s is located, and $\beta_{s}$ the site-specific effect of released salmon fry on the observed fish density. The parameter $\beta_{s}$ was drawn from a normal distribution with the hyper-mean $\theta_{\beta }$ and hyper-variance $\sigma_{\beta }^{2}$. The parameter $\varepsilon_{s,t}^{\mathrm{obs}}$ is the error term that follows a normal distribution $\mathrm{Normal}(0,\sigma_{\mathrm{obs},s}^{2})$. The inclusion of this term allows the model to account for site- and year-specific observation errors, which can be caused by ecological and/or artificial factors. When modeling the unenhanced species group, ψ equals zero (otherwise ψ=1) so the model excludes the term $\beta_{s}Fry_{w\left(s\right),t}$.

In the state model, we model temporal dynamics of fish density $n_{s,t}$ as follows:

[7] $$\begin{array}{c} \ln n_{s,t}=\xi_{1,s}+\xi_{2,s}\ln n{\;'}_{s,t-1}+\varepsilon_{s,t}^{\mathrm{state}}\\ \ln n{\;'}_{s,t-1}=\frac{\sum_{q=1}^{Q}\left(\tau^{q}\ln n_{s,t-q}\right)}{\sum_{q=1}^{Q}\tau^{q}} \end{array}$$

where $\xi_{1,s}$ is the site-specific constant at site s, $\xi_{2,s}$ the parameter characterizing an autoregressive (AR) process, and $\varepsilon_{s,t}^{\mathrm{state}}$ the process error that follows a normal distribution as $\varepsilon_{s,t}^{\mathrm{state}}∼\mathrm{Normal}(0,\sigma_{\mathrm{state},s}^{2})$. The parameters $\xi_{1,s}$ and $\xi_{2,s}$ were drawn from a multivariate normal distribution as $\xi ∼\mathrm{MVN}(\theta_{\xi },\Omega_{\xi })$, where ξ is the matrix of $\xi_{1,s}$ and $\xi_{2,s}$. Eq. **7** is structurally equivalent to a Q -order AR model; however, we expressed the part of AR parameters as a geometric-series $\tau^{q}$ to reduce the number of parameters (our model has two AR parameters $\xi_{2,s}$ and τ regardless of order Q). The parameter τ controls how fast the density influence attenuates toward the past (73). In this study, we set Q=3 given the life span of the study species (typically 1 to 3 y). We used median estimates of fish density $n_{s,t}$ to calculate the temporal CV, mean (μ), and SD (σ) for each site. For a whole community, we summed the densities of enhanced and unenhanced species. We summarized the reconstructed community dynamics in *SI Appendix*, Figs. S11–S13.

We assessed the predictive performance of our model using the Bayesian *P*-value (74), a value of which takes a range of 0 to 1 and indicates over- (~0.00), under- (~1.00), or suitable-fitting (~0.50) to the data. A Bayesian *P*-value for our state-space models was 0.50, indicating that our model specification is appropriate.

We used linear regression to quantify the impact of the intentional release on community dynamics. Although our focus is intentional release, each model included climatic and local abiotic variables to account for important environmental differences among sites. Specifically, we developed the following linear regression model taking either taxonomic richness (the number of taxa present during the observation period), mean, or SD as a response variable $y_{s}$.

[8] $$\begin{array}{c} \left\{ \begin{array}{ccc} y_{s} & ∼\mathrm{Poisson}(\lambda_{y,s}exp(\varepsilon_{\lambda ,s})) & \mathrm{for}\;\mathrm{taxonomic}\;\mathrm{richness}\\ lny_{s} & ∼\mathrm{MVN}(\theta_{y,s},\Omega_{y}) & \mathrm{for}\;\mathrm{mean}\;\mathrm{and}\;\mathrm{SD} \end{array}.\right. \end{array}$$

In the Poisson model for taxonomic richness, $exp\left(\varepsilon_{\lambda ,s}\right)$ is the error term accounting for overdispersion ($\varepsilon_{\lambda ,s}∼\mathrm{Normal}(0,\sigma_{\lambda }^{2})$). In the multivariate normal model, $y_{s}$ represents a vector of mean and SD at site s, which is modeled as a random variable drawn from a multivariate normal distribution with the variance–covariance matrix $\Omega_{y}$. The expected means $\lambda_{y,s}$ and $\theta_{y,s}$ were related to linear predictors as follows:

[9] $$\begin{array}{c} \{\begin{array}{ccc} ln\lambda_{y,s} & =\gamma_{0,w\left(s\right)}+\gamma_{1}R\;{'}_{s}+\sum_{k}\gamma_{k}x_{k,s} & \mathrm{for}\;\mathrm{taxonomic}\;\mathrm{richness}\\ \theta_{y,s} & =\gamma_{0,w\left(s\right)}+\gamma_{1}R\;{'}_{s}+\sum_{k}\gamma_{k}x_{k,s} & \mathrm{for}\;\mathrm{mean}\;\mathrm{and}\;\mathrm{SD} \end{array} . \end{array}$$

$\gamma_{0,w\left(s\right)}$ is the watershed-specific intercept ($w\left(s\right)$ refers to site s nested within watershed w) and $\gamma_{k}(k=1,2,...)$ is the $k^{\mathrm{th}}$ regression coefficient [effective release of masu salmon $R{\;'}_{s}$ (k=1) and other site-level predictors $x_{k}$ (k≥2)]. $R{\;'}_{s}$ is the product of the average yearly release at watershed w ($R_{w}$; fry + juvenile + smolt, averaged for 1999 to 2019) and the site-specific weight factor $\eta_{w,s'}$ ($s'=1,...,S_{w}+1$, where $S_{w}$ is the number of sites within watershed w):

[10] $$\begin{array}{c} \begin{array}{c} R{\;'}_{s}=\eta_{w,s\;'}R_{w}\mathrm{where}\sum_{s}^{S_{w}+1}\eta_{w,s\;'}=1 \end{array} \end{array}.$$

In Eq. **10**, $\eta_{w,s\;'}$ represents the effective proportion of total release affecting the community at site s' within watershed w. The summation of $\eta_{w,s\;'}$ over $S_{w}+1$ is assumed to be unity because a non-negligible portion of release may escape into unsurveyed areas. Since the values of $\eta_{w,s\;'}$ are not available in our dataset, we estimated them through a stochastic search while fitting the regression model to the data within a Bayesian framework (*Model Fitting*). Other site-level predictors include upstream watershed area (log-transformed), air temperature, precipitation, forest land use, and the number of observation years. The number of observation years was included as a control factor given the potential influence on the estimates of temporal community variability. Urban and agricultural land use was omitted because of either a limited value range (*SI Appendix*, Fig. S14) or a strong correlation with forest land use (*SI Appendix*, Fig. S15). The watershed-specific intercept was related to a watershed-level predictor as follows:

[11] $$\begin{array}{c} \begin{array}{cc} \gamma_{0,w} & ∼\mathrm{Normal}(\theta_{\gamma ,w},\sigma_{\gamma }^{2})\\ \theta_{\gamma ,w} & =\gamma {'}_{0}+\sum_{k}\gamma {'}_{k}x{'}_{k,w} \end{array} \end{array},$$

$\gamma {\;'}_{k}$ is the global intercept and $\gamma {\;'}_{k}$ is the regression coefficients of ocean productivity $\gamma {\;'}_{2,w}$ (chlorophyll *a* concentration; averaged for 2002 to 2019) and SD elevation within an entire watershed $\gamma {\;'}_{2,w}$. Ocean productivity was included because the majority of the observed species use marine habitats at a certain life stage (i.e., diadromous). SD elevation is a landscape-level variable that may dictate long-term flow dynamics. The parameter $\sigma_{\gamma }$ accounts for random variation among watersheds that the watershed-level predictors cannot capture.

Since the CV is an additive function of mean μ and SD σ in a log scale, we can derive the effects of environmental factors on the CV from Eq. **9**:

[12] $$\begin{array}{c} E\left(\mathrm{In}CV_{s}\right)=E\left(\mathrm{In}y_{s}^{\left(\sigma \right)}\right)-E\left(\mathrm{In}y_{s}^{\left(\mu \right)}\right) \end{array}.$$

Considering that $E(lny_{s}^{\left(\sigma \right)})=\theta_{y,s}^{\left(\sigma \right)}$ and $E(lny_{s}^{\left(\mu \right)})=\theta_{y,s}^{\left(\mu \right)}$,

[13] $$\begin{array}{c} E(lnCV_{s})=(\gamma_{0,w\left(s\right)}^{\left(\sigma \right)}-\gamma_{0,w\left(s\right)}^{\left(\mu \right)})+(\gamma_{1}^{\left(\sigma \right)}-\gamma_{1}^{\left(\mu \right)})R\;{'}_{s}+\sum_{k}(\gamma_{k}^{\left(\sigma \right)}-\gamma_{k}^{\left(\mu \right)})x_{k,s} \end{array}.$$

All predictors were standardized (mean = 0, SD = 1) before the analysis.

#### Species interaction.

We analyzed a subset of our data to estimate the strength of interspecific interactions among stream fishes. In this analysis, we focused on nine sites with >16 y of observations and no intentional release of masu salmon. Further, at each site, we confined our analysis to species that occurred more than four times. These additional constraints were necessary to estimate parameters in a complex multi-species model within a state-space modeling framework.

We fitted the following state-space model to the data for each site separately. In the observation model, we modeled fish abundance $N_{i,t}$ for species i at year t as follows:

[14] $$\begin{array}{c} \begin{array}{cc} N_{i,t} & ∼\mathrm{Poisson}(\lambda_{i,t}A_{t})\\ \lambda_{s,t} & =n_{i,t}exp\left(\varepsilon_{i,t}^{\mathrm{obs}}\right) \end{array} \end{array},$$

where $\varepsilon_{i,t}^{\mathrm{obs}}∼\mathrm{Normal}(0,\sigma_{\mathrm{obs},i})$. In the state model, we described a multi-species Ricker model for fish density $n_{i,t}$:

[15] $$\begin{array}{c} lnn_{i,t}=lnn_{i,t-1}+r_{i}-\sum_{j=1}^{S}\alpha_{ij}^{\prime }n_{j,t-1}+\varepsilon_{i,t}^{\mathrm{state}} \end{array}.$$

Eq. **15** is equivalent to Eq. **1** except the scale of competition coefficients $\alpha_{\mathrm{ij}}^{\prime }$ ($\frac{\alpha_{\mathrm{ij}}^{\prime }}{\alpha_{\mathrm{ii}}^{\prime }}=\alpha_{\mathrm{ij}}$, where $\alpha_{\mathrm{ii}}^{\prime }=\frac{r_{i}}{K_{i}}$). We used sparse priors for $\alpha_{\mathrm{ij}}^{\prime }$ to reduce the risk of over-fitting (75):

[16] $$\begin{array}{c} \begin{array}{c} \alpha_{\mathrm{ij}}^{\prime }∼\mathrm{Half}-\mathrm{normal}(0,\sigma_{\alpha ,ij}^{2})\\ \sigma_{\alpha ,ij}^{2}=z_{\mathrm{ij}}c_{1}+(1-z_{\mathrm{ij}})c_{0} \end{array} \end{array}.$$

In Eq. **16**, the latent variable $z_{\mathrm{ij}}$ is drawn from a Bernoulli distribution and controls the prior for $\alpha_{\mathrm{ij}}^{\prime }$. We used $c_{1}=1$ and $c_{0}=0.01$ so that $\alpha_{\mathrm{ij}}^{\prime }$ is freely estimated when $z_{\mathrm{ij}}$ equals one ($\sigma_{\alpha }^{2}=1$). Otherwise, the probability density of $\alpha {'}_{\mathrm{ij}}$ would be concentrated around zero ($\sigma_{\alpha }^{2}=0.01$), effectively omitting the parameter from the model (75). We assigned different “inclusion” probabilities to diagonal (intraspecific competition) and off-diagonal elements (interspecific competition) as $z_{\mathrm{ii}}∼\mathrm{Bernoulli}\left(p_{\alpha }^{intra}\right)$ and $z_{ij,i\neq j}∼\mathrm{Bernoulli}\left(p_{\alpha }^{inter}\right)$.

We assumed that environmental noise $\varepsilon_{i,t}^{\mathrm{state}}$ is correlated among species by introducing the variance–covariance matrix $\Omega_{\varepsilon }$. The matrix $\Omega_{\varepsilon }$ was modeled with the aid of latent factors $\zeta_{t,d}$ and factor loadings $\delta_{d,i}$:

[17] $$\begin{array}{c} \varepsilon_{i,t}^{\mathrm{state}}=\sum_{d=1}^{D}\zeta_{t,d}\delta_{d,i}+I_{\mathrm{ij}}\pi_{i,t} \end{array},$$

where D is the number of latent factors, $\pi_{i,t}∼\mathrm{Normal}(0,\sigma_{\pi ,i}^{2})$, and $I_{\mathrm{ij}}$ is the identity matrix ($I_{\mathrm{ii}}=1$ and $I_{ij,i\neq j}=0$). With this formulation, the matrix $\Omega_{\varepsilon }$ can be described as $\Omega_{\varepsilon }=\Delta^{T}\Delta +\mathrm{diag}\left(\sigma_{\pi ,i}^{2}\right)$, where Δ is the matrix of factor loadings $\delta_{d,i}$. This model structure with multiplicative gamma priors on factor loadings (76) has the merit of the reduced number of parameters while capturing the correlated nature of species responses to environmental fluctuations (77). We set D=2 in our model. We reported the estimates of $\alpha_{\mathrm{ij}}$, so the values are comparable to those used in the theoretical analysis.

#### Model fitting.

We fitted the models to the data using Just Another Gibbs Sampler (JAGS) version 4.1.0 through *runjags* package version 2.2.0-2 in R (78). We assigned vague or weakly informative priors to parameters (*SI Appendix*, Table S3). Four Markov chain Monte Carlo (MCMC) chains were run until parameter estimates converged. The total MCMC iterations ranged from 15,000 to 35,000, in which MCMC samples were saved every 40 steps for the calculation of posterior distributions after the initial 5,000 burn-in period. Convergence was assessed by examining whether the $\hat{R}$ indicator of each parameter approached <1.1 (79). Data manipulation and analysis were performed in R version 4.2.1 (80). Parameter estimates were summarized in *SI Appendix*, Tables S4–S8.

## Supplementary Material

Appendix 01 (PDF)

## Data Availability

Data and codes are available at https://github.com/aterui/public-proj_fishery-stability (81).
