# Supplementary material for: Intentional release of native species undermines ecological stability
Source: Proc Natl Acad Sci U S A. 2023 Feb 7;120(7):e2218044120. doi: 10.1073/pnas.2218044120 (PMC9963293; doi:10.1073/pnas.2218044120)
Supplement: Supplementary file 1 — Appendix 01 (PDF) [file pnas.2218044120.sapp.pdf]

PNAS

Supplementary Materials for:

Intentional release of native species undermines ecological stability

Akira Terui, Hirokazu Urabe, Masayuki Senzaki, Bungo Nishizawa

Correspondence to: Akira Terui hanabi0111@gmail.com

This PDF file includes:

- Supplementary text
- Tables S1 – 8
- Figures S1 – S17
- References

Contents

1 Supplementary Text **1**
- 1.1 Two-species model . . . 1
- 1.2 Fish sampling . . . 1

2 Supplementary Tables **2**
- 2.1 Simulation parameter . . . 2
- 2.2 Observed species . . . 3
- 2.3 Priors . . . 4
- 2.4 Parameter estimates for the state-space autoregressive model . . . 5
- 2.5 Parameter estimates for the state-space Ricker model . . . 6
- 2.6 Parameter estimates for the regression model (whole community) . . . 7
- 2.7 Parameter estimates for the regression model (masu salmon) . . . 8
- 2.8 Parameter estimates for the regression model (unenhanced species) . . . 9

3 Supplementary Figures **10**
- 3.1 Theoretical prediction (two-species community with weak competition) . . . 10
- 3.2 Theoretical prediction (two-species community with strong competition) . . . 11
- 3.3 Theoretical prediction ($r_1 = 0.5$, $K = 100$) . . . 12
- 3.4 Theoretical prediction ($r_1 = 1.5$, $K = 100$) . . . 13
- 3.5 Theoretical prediction ($r_1 = 2.5$, $K = 100$) . . . 14
- 3.6 Theoretical prediction ($r_1 = 3.5$, $K = 100$) . . . 15
- 3.7 Theoretical prediction ($r_1 = 0.5$, $K = 400$) . . . 16
- 3.8 Theoretical prediction ($r_1 = 1.5$, $K = 400$) . . . 17
- 3.9 Theoretical prediction ($r_1 = 2.5$, $K = 400$) . . . 18
- 3.10 Theoretical prediction ($r_1 = 3.5$, $K = 400$) . . . 19
- 3.11 Temporal dynamics of stream fish communities (whole community) . . . 20
- 3.12 Temporal dynamics of stream fish communities (masu salmon) . . . 21
- 3.13 Temporal dynamics of stream fish communities (unenhanced species) . . . 22
- 3.14 Environmental variables . . . 23
- 3.15 Correlation plot . . . 24
- 3.16 Co-occurrence matrix . . . 25
- 3.17 Empirical estimates of competition coefficients . . . 26

References **27**

1 Supplementary Text

1.1 Two-species model

The analysis of a two-species community revealed the distinct parameter spaces predicting a stabilizing, neutral, or destabilizing effect of intentional release. The left column in **Figure S1A** shows the predictions in scenarios with no environmental stochasticity ($\sigma_\varepsilon = 0$) and weak competition ($\bar{\alpha} = 0.25$). For lower values of intrinsic growth rate r_i (< 1.5), intentional release had neutral (with higher K) to destabilizing effects (with lower K) on community dynamics. In the meantime, stabilization with intentional release occurred for higher values of r_i (≥ 1.5) by dampening limit cycles or chaos (**Figure S**1), which is reflected in decreased SD σ (**Figure S**1). However, the long-term mean of community density μ consistently decreased with intentional release in almost all cases except for only a few combinations of r_i and K (**Figure S**1).

Environmental stochasticity did not change the qualitative patterns of deterministic simulations; however, it greatly expanded the parameter space with destabilizing effects of intentional release (the right columns in **Figure S**1**A and B**). In particular, destabilizing effects were more broadly observed when intrinsic growth rates were low.

Increasing the competition coefficient $\bar{\alpha}$ shifted the region with the destabilizing effects to higher carrying capacities ($\bar{\alpha} = 0.5$, **Figure S**2); otherwise, general patterns were similar to the weak competition scenario.

1.2 Fish sampling

The monitoring program employed a two-pass sampling method at all sampling sites. In each pass, a combination of electrofishing and cast net was used to effectively catch both benthic and water-column species in a given area. The number of cast-net throws was determined as the sampled area divided by 4.5 m^2 (the area covered by a cast net). Thus, this sampling method has a constant sampling effort per unit area across sites.

2 Supplementary Tables

2.1 Simulation parameter

Table S1: Parameter values used in the whole community simulation.

Parameter	Interpretation	Value
S	Number of species	10
r_1	Intrinsic growth rate of an enhanced species	0.5, 1.5, 2.5, 3.5
r_{max}	Maximum intrinsic growth rate of unenhanced species	2.5
$\bar{\alpha}$	Average strength of interspecific competition	0.25, 0.50
K	Carrying capacity	100, 400
σ_ε	Environmental variability	0.5
f_R	Relative fitness of captive-bred individuals	0.5, 1.0

2.2 Observed species

Table S2: Fish species found in the study watersheds. Body size is coded as follows: maximum total length (TL) < 100 mm (S), $100 \leq \text{TL} \leq 200$ (M), and $\text{TL} > 200$ (L).

Taxon	Current preference	Trophic guild	Vertical position	Spawning substrate	Body size
Barbatula oreas	fast	omnivore	bottom	various	M
Cottus spp.	fast	invertivore-piscivore	bottom	mineral substrate	M
Gasterosteus spp.	slow	invertivore	water column	vegetation	M
Gymnogobius spp.	various	invertivore	bottom	mineral substrate	M
Lethenteron spp.	slow	detritivore	bottom	mineral substrate	L
Luciogobius guttatus	slow	invertivore	bottom	mineral substrate	S
Misgurnus anguillicaudatus	stagnant	omnivore	bottom	various	L
Oncorhynchus masou masou	fast	invertivore-piscivore	water column	mineral substrate	L
Oncorhynchus mykiss	fast	invertivore-piscivore	water column	mineral substrate	L
Parahucho perryi	slow	invertivore-piscivore	water column	mineral substrate	M
Plecoglossus altivelis altivelis	fast	algaevore	water column	mineral substrate	L
Pseudaspius spp.	slow	omnivore	water column	various	L
Pseudorasbora spp.	stagnant	omnivore	water column	various	M
Pungitius spp.	slow	invertivore	water column	vegetation	S
Rhinogobius spp.	various	omnivore	bottom	mineral substrate	S
Rhynchocypris lagowskii steindachneri	stagnant	omnivore	water column	various	L
Rhynchocypris percnura sachalinensis	stagnant	omnivore	water column	vegetation	L
Salvelinus leucomaenis leucomaenis	fast	invertivore-piscivore	water column	mineral substrate	L
Salvelinus malma krascheninnikovi	fast	invertivore	water column	mineral substrate	L
Tridentiger brevispinis	slow	omnivore	bottom	mineral substrate	M

Note: Trait information is based on the literature (1–5) and expert knowledge.

2.3 Priors

Table S 3: Prior distributions used in the Bayesian models. The parameter $\boldsymbol{o}$ is a vector of prior scales ($\boldsymbol{o} = 2.5, ..., 2.5$).

Model	Parameter	Prior
AR model	θ_β	Normal(0, 10)
	θ_{ξ_1}	Normal(0, 10)
	θ_{ξ_2}	Normal(1, 10)
	τ	Unif(0, 1)
	σ_β	Half-t(0, 2.5, 6)
	$\sigma_{\text{obs},s}$	Half-t(0, 2.5, 6)
	$\sigma_{\text{state},s}$	Half-t(0, 2.5, 6)
	$\boldsymbol{\Omega_\xi}$	Scaled Inv-Wishart$_2(\boldsymbol{o})$
	$\ln n_{s,1-3}$	Half-t(0, 2.5, 6)
Ricker model	$\sigma_{\text{obs},i}$	Half-t(0, 2.5, 6)
	$\sigma_{\text{state},i}$	Half-t(0, 2.5, 6)
	σ_λ	Half-t(0, 2.5, 6)
	σ_γ	Half-t(0, 2.5, 6)
	$\sigma_{\epsilon,i}$	Half-t(0, 2.5, 6)
	$\boldsymbol{\Omega_\varepsilon}$	Scaled Inv-Wishart$_2(\boldsymbol{o})$
	p_α^{intra}	Beta(1, 1)
	p_α^{inter}	Beta(1, 1)
	$\zeta_{t,d}$	Normal(0, 1)
	$\delta_{d,i}$	Multiplicative gamma prior ($\nu = 3$, $a_1 = 2$, $a_2 = 3$)
	$\ln n_{i,1}$	Half-t(-2, 2, 6)
Regression	γ	Normal(0, 10)
	γ'	Normal(0, 10)
	σ_λ	Half-t(0, 2.5, 4)
	σ_γ	Half-t(0, 2.5, 4)
	$\boldsymbol{\eta_w}$ ($\boldsymbol{\eta_w} = \eta_{w,1}, ..., \eta_{w,S_w}$)	Dirichlet(1,...,1)
	$\boldsymbol{\Omega_y}$	Scaled Inv-Wishart$_2(\boldsymbol{o})$

Note: see Durante (6) for multiplicative gamma priors.

2.4 Parameter estimates for the state-space autoregressive model

Table S4: Median estimates for key parameters of the state-space autoregressive model. Site-specific parameters were excluded due to a large number of parameters.

Group	Parameter	Estimate	95% CI
Enhanced (masu salmon)	θ_β	0.58	0.36 to 0.87
	σ_β	0.58	0.34 to 0.89
	$\theta_{\xi 1}$	−0.33	−0.51 to − 0.21
	$\theta_{\xi 2}$	0.82	0.73 to 0.89
	τ	0.73	0.45 to 0.97
Unenhanced	$\theta_{\xi 1}$	−0.94	−1.28 to − 0.56
	$\theta_{\xi 2}$	0.53	0.38 to 0.71
	τ	0.64	0.26 to 0.96

2.5 Parameter estimates for the state-space Ricker model

Table S5: Median estimates for key parameters of the state-space Ricker model. Site-specific parameters were excluded due to a large number of parameters.

Parameter	Site	Estimate	95% CI
$p_{\alpha}^{\text{intra}}$	Atsuta2	0.63	0.04 to 0.98
	Atsuta3	0.67	0.07 to 0.98
	Atsuta4	0.61	0.04 to 0.98
	Ookamotsu1	0.68	0.06 to 0.98
	Ookamotsu2	0.62	0.02 to 0.99
	Shakotan1	0.69	0.06 to 0.99
	Shakotan3	0.75	0.19 to 0.99
	Shakotan4	0.77	0.17 to 0.99
	Shimonaefutoro1	0.68	0.07 to 0.99
$p_{\alpha}^{\text{inter}}$	Atsuta2	0.38	0.02 to 0.94
	Atsuta3	0.47	0.03 to 0.96
	Atsuta4	0.48	0.04 to 0.96
	Ookamotsu1	0.61	0.04 to 0.98
	Ookamotsu2	0.51	0.02 to 0.97
	Shakotan1	0.39	0.02 to 0.95
	Shakotan3	0.38	0.03 to 0.96
	Shakotan4	0.36	0.01 to 0.96
	Shimonaefutoro1	0.48	0.02 to 0.97

2.6 Parameter estimates for the regression model (whole community)

Table S6: Parameter estimates for the regression model (whole community). Median estimates and standard errors (SEs) of regression coefficients are shown. Pr(> 0) and Pr(< 0) represent the proportion of positive and negative estimates in MCMC samples, respectively (i.e., posterior probability).

Response	Variable	Estimate	SE	Pr(> 0)	Pr(< 0)
CV	Intercept	−0.91	0.06	0.00	1.00
	Effective release	0.18	0.05	1.00	0.00
	Watershed area	0.11	0.04	1.00	0.00
	Air temperature	−0.05	0.06	0.21	0.79
	Precipitation	−0.04	0.06	0.23	0.77
	Forest fraction	0.03	0.09	0.65	0.35
	Number of observations	0.15	0.06	0.99	0.01
	Ocean productivity	−0.01	0.08	0.45	0.55
	SD elevation	0.04	0.06	0.75	0.25
Taxonomic richness	Intercept	1.74	0.05	1.00	0.00
	Effective release	−0.09	0.06	0.04	0.96
	Watershed area	0.08	0.05	0.96	0.04
	Air temperature	0.08	0.06	0.93	0.07
	Precipitation	−0.05	0.06	0.18	0.82
	Forest fraction	−0.16	0.08	0.01	0.99
	Number of observations	0.04	0.05	0.79	0.21
	Ocean productivity	−0.16	0.07	0.02	0.98
	SD elevation	−0.09	0.05	0.04	0.96
Mean μ	Intercept	−1.02	0.06	0.00	1.00
	Effective release	−0.20	0.06	0.00	1.00
	Watershed area	−0.16	0.05	0.00	1.00
	Air temperature	0.18	0.07	1.00	0.00
	Precipitation	−0.05	0.06	0.19	0.81
	Forest fraction	0.06	0.09	0.75	0.25
	Number of observations	−0.00	0.06	0.48	0.52
	Ocean productivity	−0.14	0.08	0.04	0.96
	SD elevation	−0.09	0.06	0.09	0.91
SD σ	Intercept	−1.94	0.06	0.00	1.00
	Effective release	−0.02	0.07	0.39	0.61
	Watershed area	−0.06	0.06	0.19	0.81
	Air temperature	0.14	0.07	0.96	0.04
	Precipitation	−0.09	0.07	0.09	0.91
	Forest fraction	0.09	0.10	0.84	0.16
	Number of observations	0.15	0.07	0.99	0.01
	Ocean productivity	−0.15	0.09	0.05	0.95
	SD elevation	−0.04	0.07	0.25	0.75

2.7 Parameter estimates for the regression model (masu salmon)

Table S 7: Parameter estimates for the regression model (masu salmon). Median estimates and standard errors (SEs) of regression coefficients are shown. Pr(> 0) and Pr(< 0) represent the proportion of positive and negative estimates in MCMC samples, respectively (i.e., posterior probability).

Response	Variable	Estimate	SE	Pr(> 0)	Pr(< 0)
Mean μ	Intercept	-1.86	0.11	0.00	1.00
	Effective release	-0.13	0.09	0.08	0.92
	Watershed area	-0.16	0.08	0.02	0.98
	Air temperature	0.10	0.11	0.80	0.20
	Precipitation	-0.10	0.10	0.17	0.83
	Forest fraction	0.09	0.16	0.70	0.30
	Number of observations	0.13	0.11	0.90	0.10
	Ocean productivity	-0.07	0.15	0.32	0.68
	SD elevation	-0.05	0.12	0.32	0.68
SD σ	Intercept	-2.42	0.09	0.00	1.00
	Effective release	0.01	0.10	0.56	0.44
	Watershed area	-0.08	0.09	0.18	0.81
	Air temperature	0.03	0.10	0.62	0.38
	Precipitation	-0.02	0.10	0.43	0.57
	Forest fraction	0.22	0.13	0.96	0.04
	Number of observations	0.23	0.10	0.99	0.01
	Ocean productivity	-0.13	0.12	0.15	0.85
	SD elevation	-0.02	0.10	0.41	0.59

2.8 Parameter estimates for the regression model (unenhanced species)

Table S8: Parameter estimates for the regression model (unenhanced species). Median estimates and standard errors (SEs) of regression coefficients are shown. Pr(> 0) and Pr(< 0) represent the proportion of positive and negative estimates in MCMC samples, respectively (i.e., posterior probability).

Response	Variable	Estimate	SE	Pr(> 0)	Pr(< 0)
Mean μ	Intercept	−2.03	0.07	0.00	1.00
	Effective release	−0.29	0.08	0.00	1.00
	Watershed area	−0.29	0.07	0.00	1.00
	Air temperature	0.24	0.09	1.00	0.00
	Precipitation	0.05	0.08	0.70	0.30
	Forest fraction	−0.16	0.12	0.08	0.92
	Number of observations	−0.04	0.08	0.29	0.71
	Ocean productivity	−0.34	0.11	0.00	1.00
	SD elevation	−0.18	0.08	0.01	0.99
SD σ	Intercept	−2.89	0.08	0.00	1.00
	Effective release	−0.10	0.08	0.09	0.91
	Watershed area	−0.18	0.08	0.01	0.99
	Air temperature	0.26	0.09	1.00	0.00
	Precipitation	−0.06	0.09	0.24	0.76
	Forest fraction	−0.10	0.12	0.21	0.79
	Number of observations	0.14	0.08	0.95	0.04
	Ocean productivity	−0.27	0.11	0.01	0.99
	SD elevation	−0.14	0.08	0.04	0.96

3 Supplementary Figures

3.1 Theoretical prediction (two-species community with weak competition)

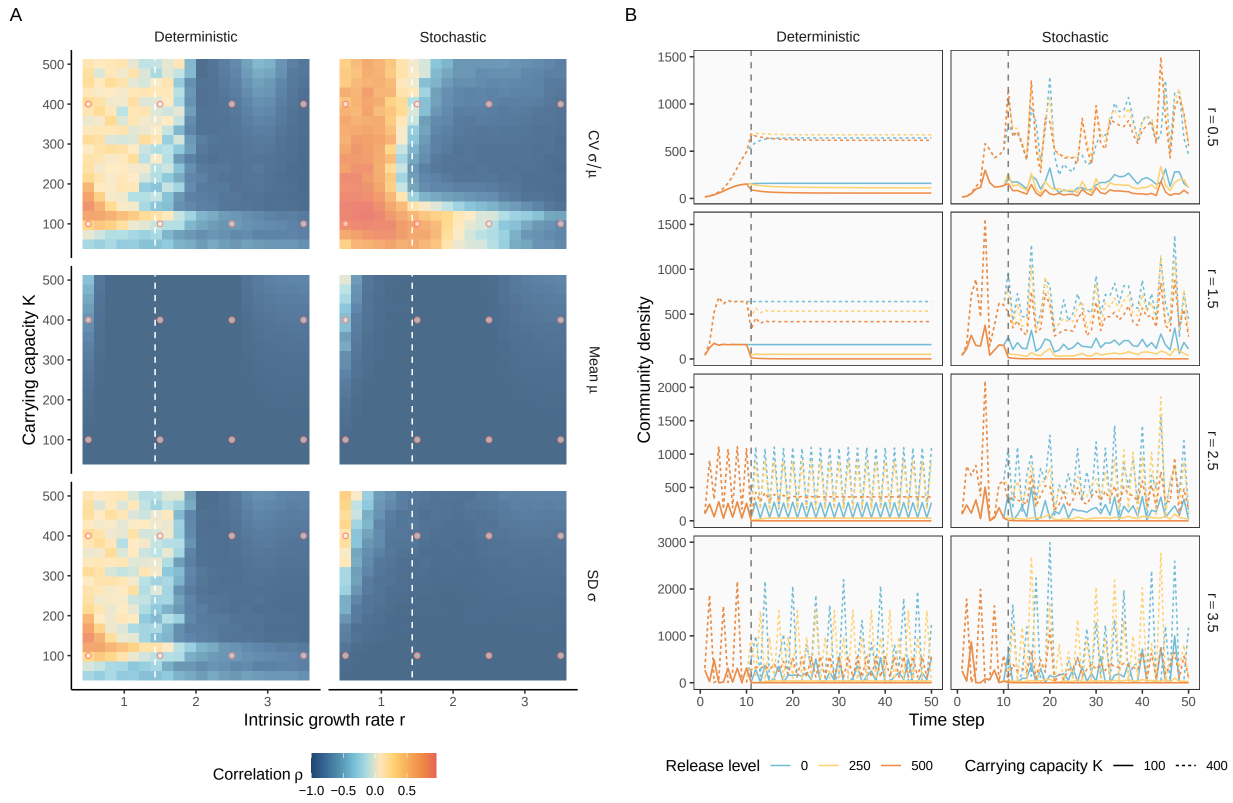

Figure S1: Theoretical predictions in a two-species community. **(A)** Spearman's rank correlations between intentional release and community dynamics in a weak competition scenario ($\bar{\alpha} = 0.25$). Rows distinguish different summary statistics of community dynamics (top: CV, middle: mean, bottom: SD) while columns distinguish deterministic ($\sigma_\varepsilon = 0$) and stochastic simulations ($\sigma_\varepsilon = 0.5$). Cells indicate 400 combinations of carrying capacity and intrinsic growth rate (20 values each). Colors are proportional to Spearman's rank correlations. Dots indicate parameter combinations that are used in the whole community simulation and Panel B. Broken vertical lines denote the average value of r_i (1.43) for salmonid species (7). **(B)** Examples of community dynamics with and without intentional release. Community dynamics diverge at broken vertical lines, the timestep at which intentional release begins. Rows distinguish intrinsic growth rates, while columns distinguish deterministic and stochastic simulations. Colors indicate release levels, and line types differ in carrying capacities.

3.2 Theoretical prediction (two-species community with strong competition)

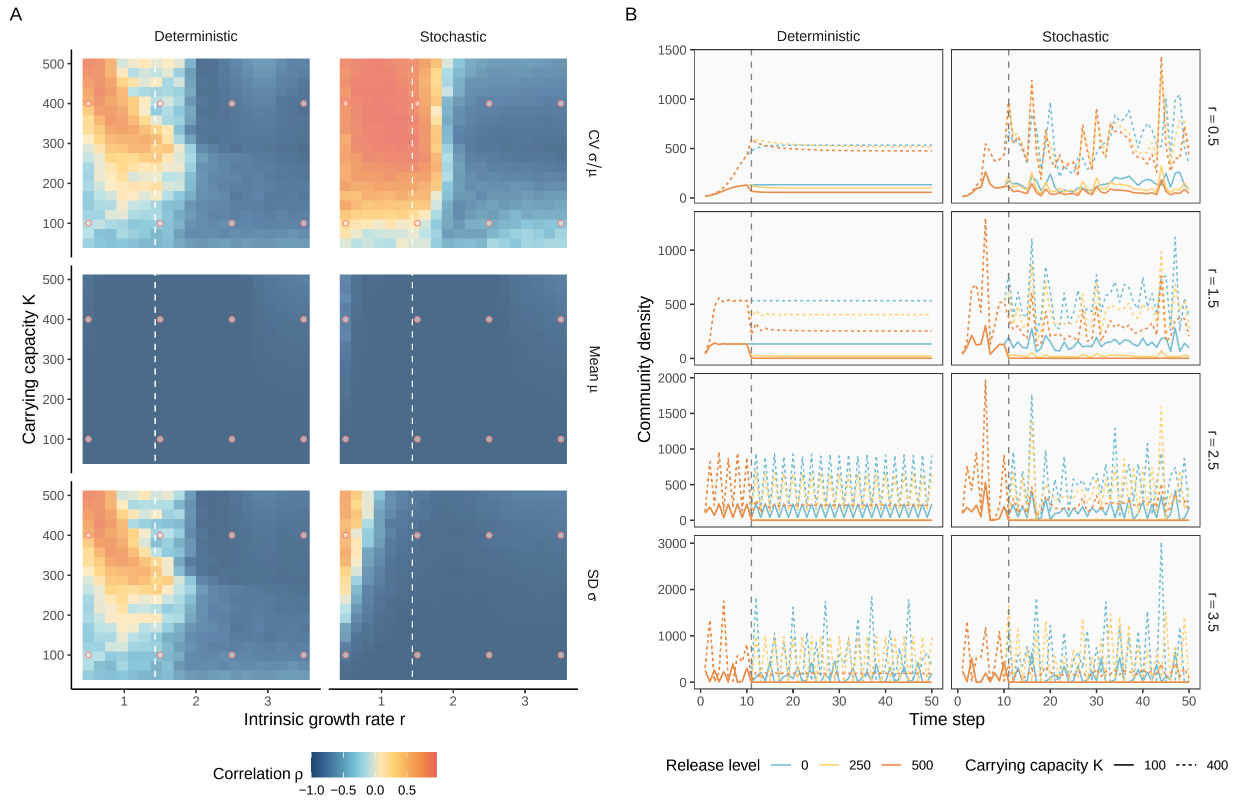

Figure S2: Theoretical predictions in a two-species community. **(A)** Spearman's rank correlations between intentional release and community dynamics in a strong competition scenario ($\bar{\alpha} = 0.5$). Rows distinguish different summary statistics of community dynamics (top: CV, middle: mean, bottom: SD) while columns distinguish deterministic ($\sigma_\varepsilon = 0$) and stochastic simulations ($\sigma_\varepsilon = 0.5$). Cells indicate 400 combinations of carrying capacity and intrinsic growth rate (20 values each). Colors are proportional to Spearman's rank correlations. Dots indicate parameter combinations that are used in the whole community simulation and Panel B. Broken vertical lines denote the average value of r_i (1.43) for salmonid species (7). **(B)** Examples of community dynamics with and without intentional release. Community dynamics diverge at broken vertical lines, the timestep at which intentional release begins. Rows distinguish intrinsic growth rates, while columns distinguish deterministic and stochastic simulations. Colors indicate release levels, and line types differ in carrying capacities.

3.3 Theoretical prediction (r_1 = 0.5, K = 100)

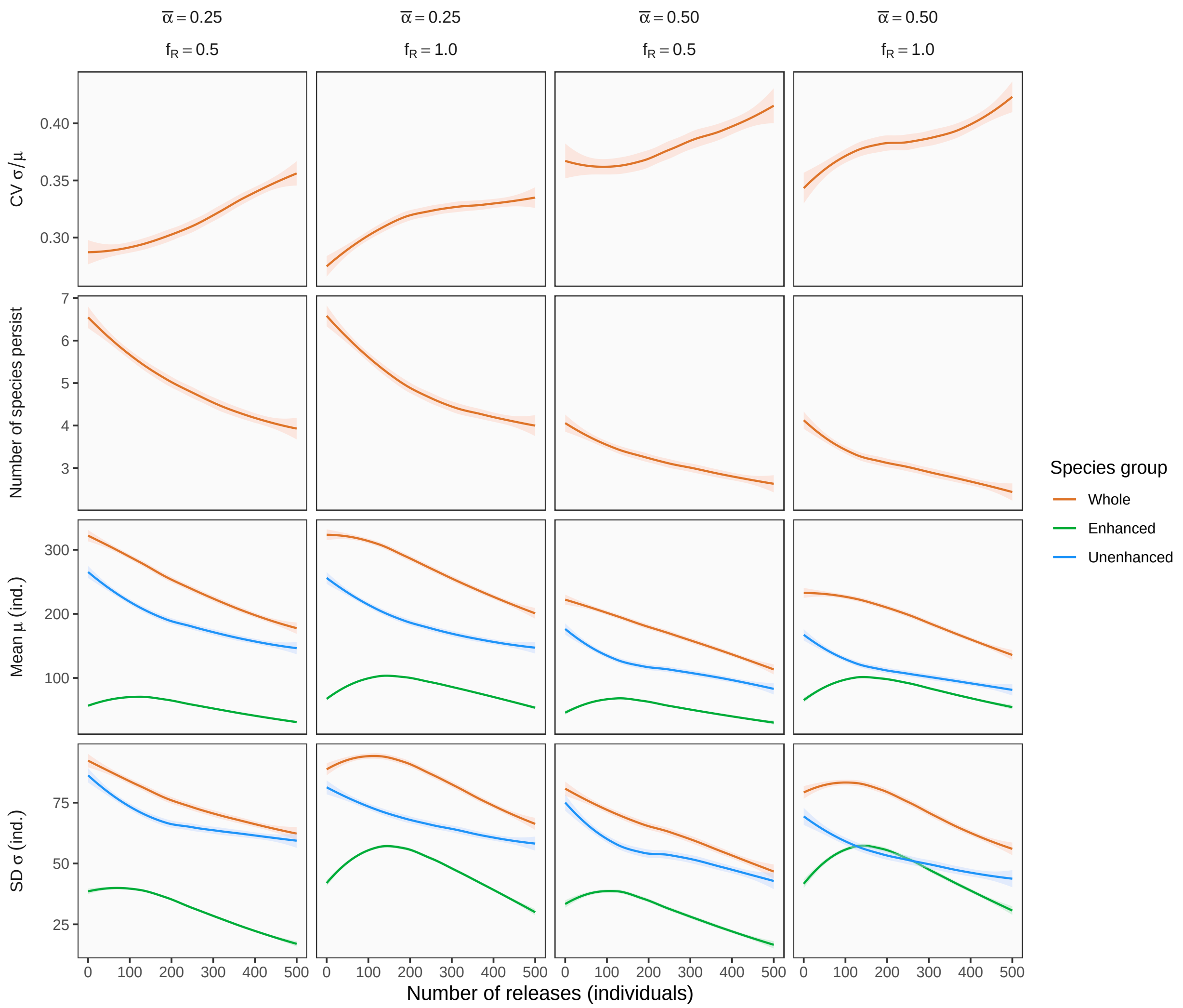

Figure S3: Theoretical predictions for the release effect in a ten-species community. Rows represent different response variables, and columns show distinct simulation scenarios with different strengths of interspecific competition ($\bar{\alpha}$) and relative fitness of captive-bred individuals (f_R). Lines are loess curves (mean) with shades indicating associated 95% confidence intervals. Other parameters are: number of species $S = 10$; intrinsic growth rate of an enhanced species $r_1 = 0.5$; maximum intrinsic growth rate of unenhanced species $r_{\max} = 2.5$; environmental stochasticity $\sigma_\varepsilon = 0.5$; carrying capacity $K = 100$.

3.4 Theoretical prediction (r_1 = 1.5, K = 100)

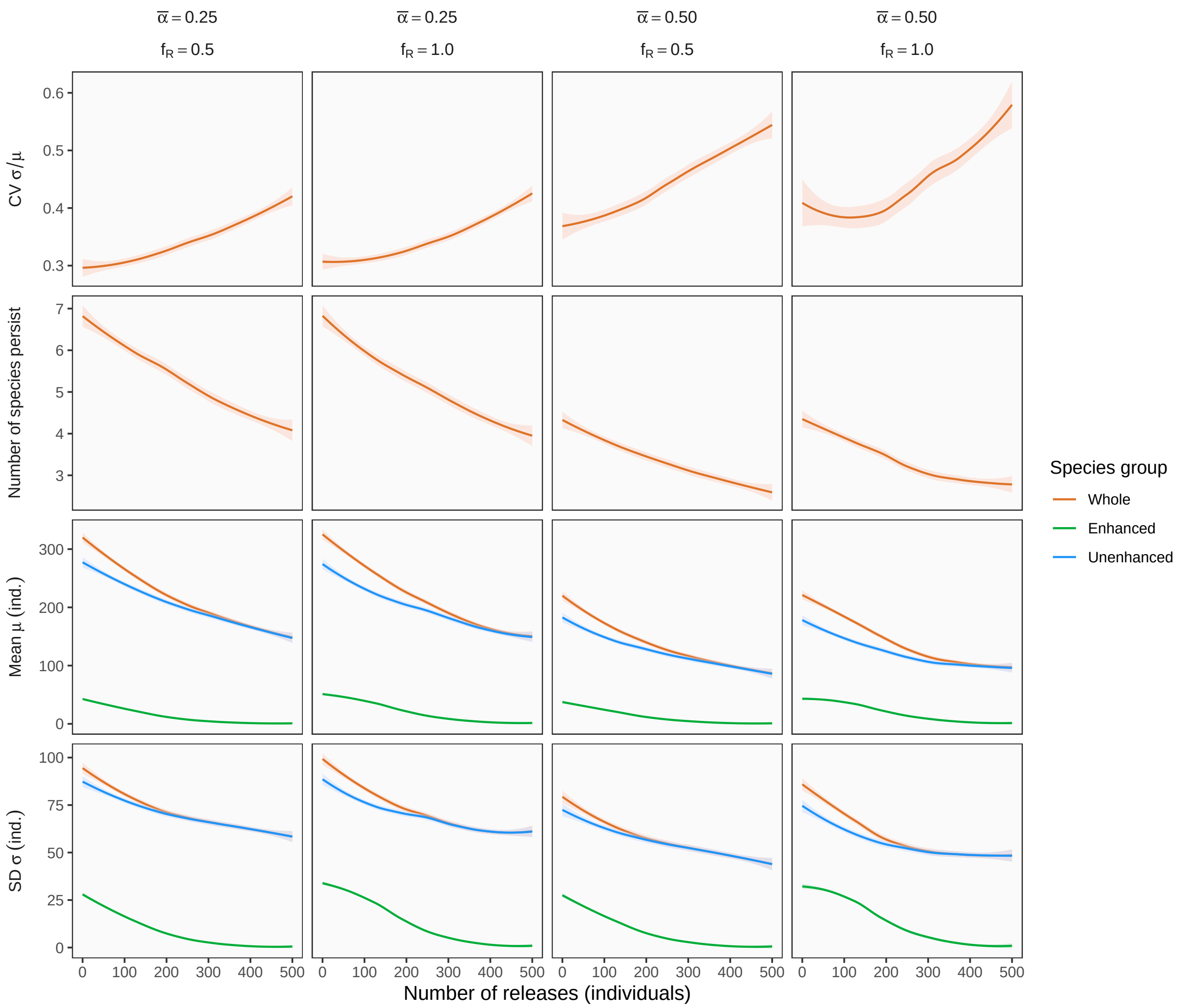

Figure S4: Theoretical predictions for the release effect in a ten-species community. Rows represent different response variables, and columns show distinct simulation scenarios with different strengths of interspecific competition ($\bar{\alpha}$) and relative fitness of captive-bred individuals (f_R). Lines are loess curves (mean) with shades indicating associated 95% confidence intervals. Other parameters are: number of species S = 10; intrinsic growth rate of an enhanced species r_1 = 1.5; maximum intrinsic growth rate of unenhanced species r_{max} = 2.5; environmental stochasticity σ_ε = 0.5; carrying capacity K = 100.

3.5 Theoretical prediction (r_1 = 2.5, K = 100)

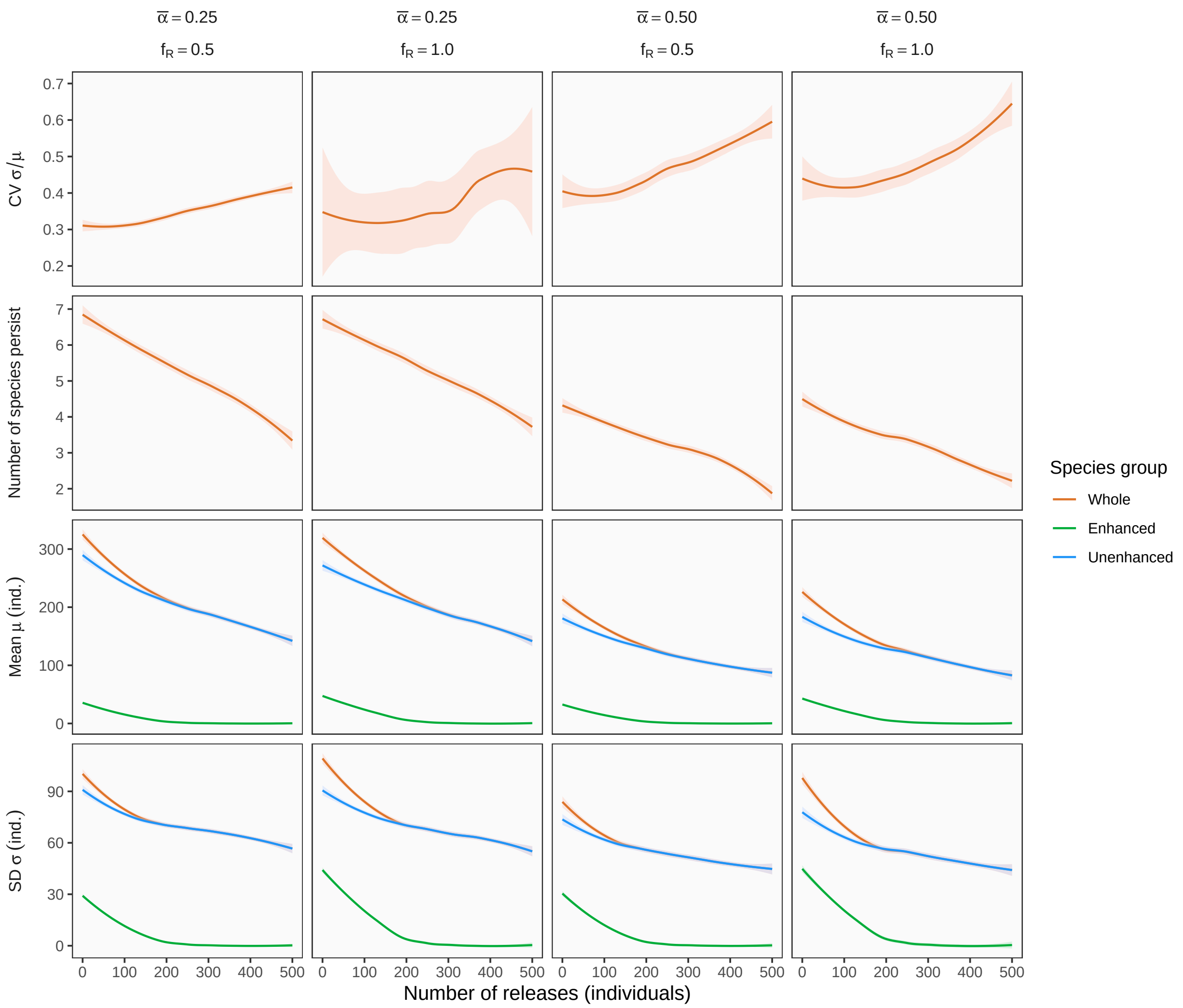

Figure S5: Theoretical predictions for the release effect in a ten-species community. Rows represent different response variables, and columns show distinct simulation scenarios with different strengths of interspecific competition ($\bar{\alpha}$) and relative fitness of captive-bred individuals (f_R). Lines are loess curves (mean) with shades indicating associated 95% confidence intervals. Other parameters are: number of species $S = 10$; intrinsic growth rate of an enhanced species $r_1 = 2.5$; maximum intrinsic growth rate of unenhanced species $r_{max} = 2.5$; environmental stochasticity $\sigma_\varepsilon = 0.5$; carrying capacity $K = 100$.

3.6 Theoretical prediction (r_1 = 3.5, K = 100)

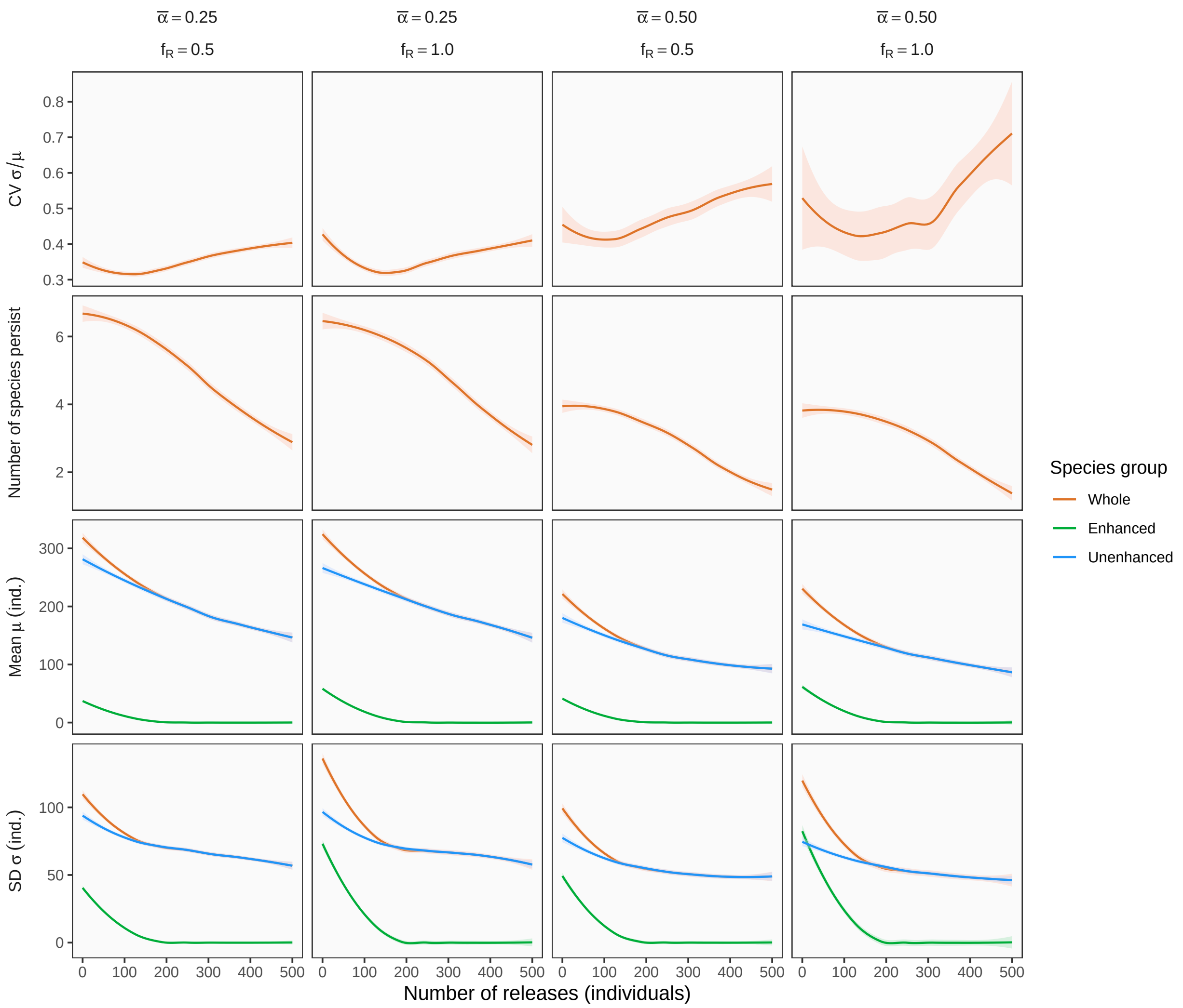

Figure S6: Theoretical predictions for the release effect in a ten-species community. Rows represent different response variables, and columns show distinct simulation scenarios with different strengths of interspecific competition ($\bar{\alpha}$) and relative fitness of captive-bred individuals (f_R). Lines are loess curves (mean) with shades indicating associated 95% confidence intervals. Other parameters are: number of species S = 10; intrinsic growth rate of an enhanced species r_1 = 3.5; maximum intrinsic growth rate of unenhanced species $r_{\max}$ = 2.5; environmental stochasticity σ_ε = 0.5; carrying capacity K = 100.

3.7 Theoretical prediction (r_1 = 0.5, K = 400)

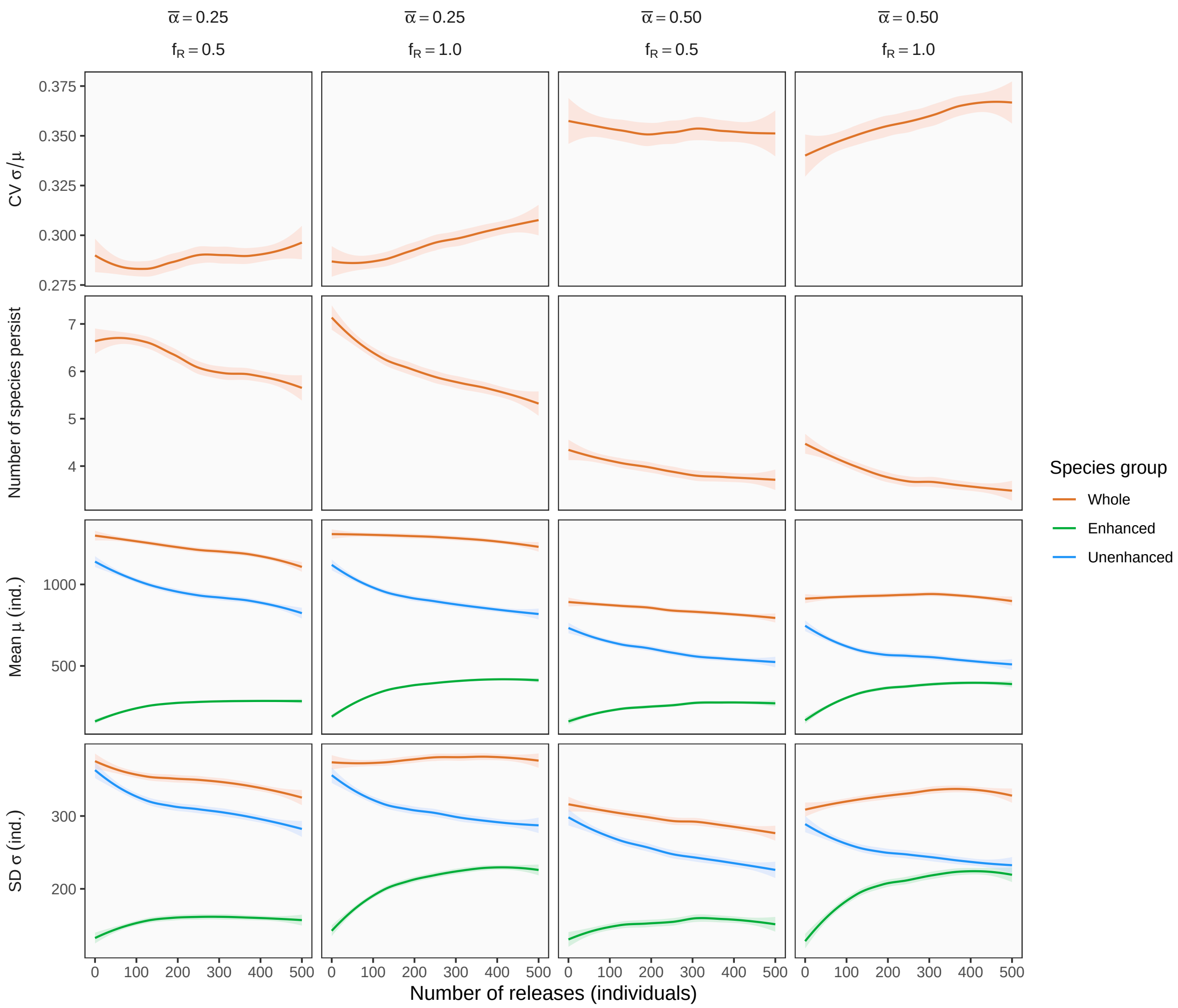

Figure S7: Theoretical predictions for the release effect in a ten-species community. Rows represent different response variables, and columns show distinct simulation scenarios with different strengths of interspecific competition ($\bar{\alpha}$) and relative fitness of captive-bred individuals (f_R). Lines are loess curves (mean) with shades indicating associated 95% confidence intervals. Other parameters are: number of species S = 10; intrinsic growth rate of an enhanced species r_1 = 0.5; maximum intrinsic growth rate of unenhanced species $r_{\max}$ = 2.5; environmental stochasticity σ_ε = 0.5; carrying capacity K = 400.

3.8 Theoretical prediction (r_1 = 1.5, K = 400)

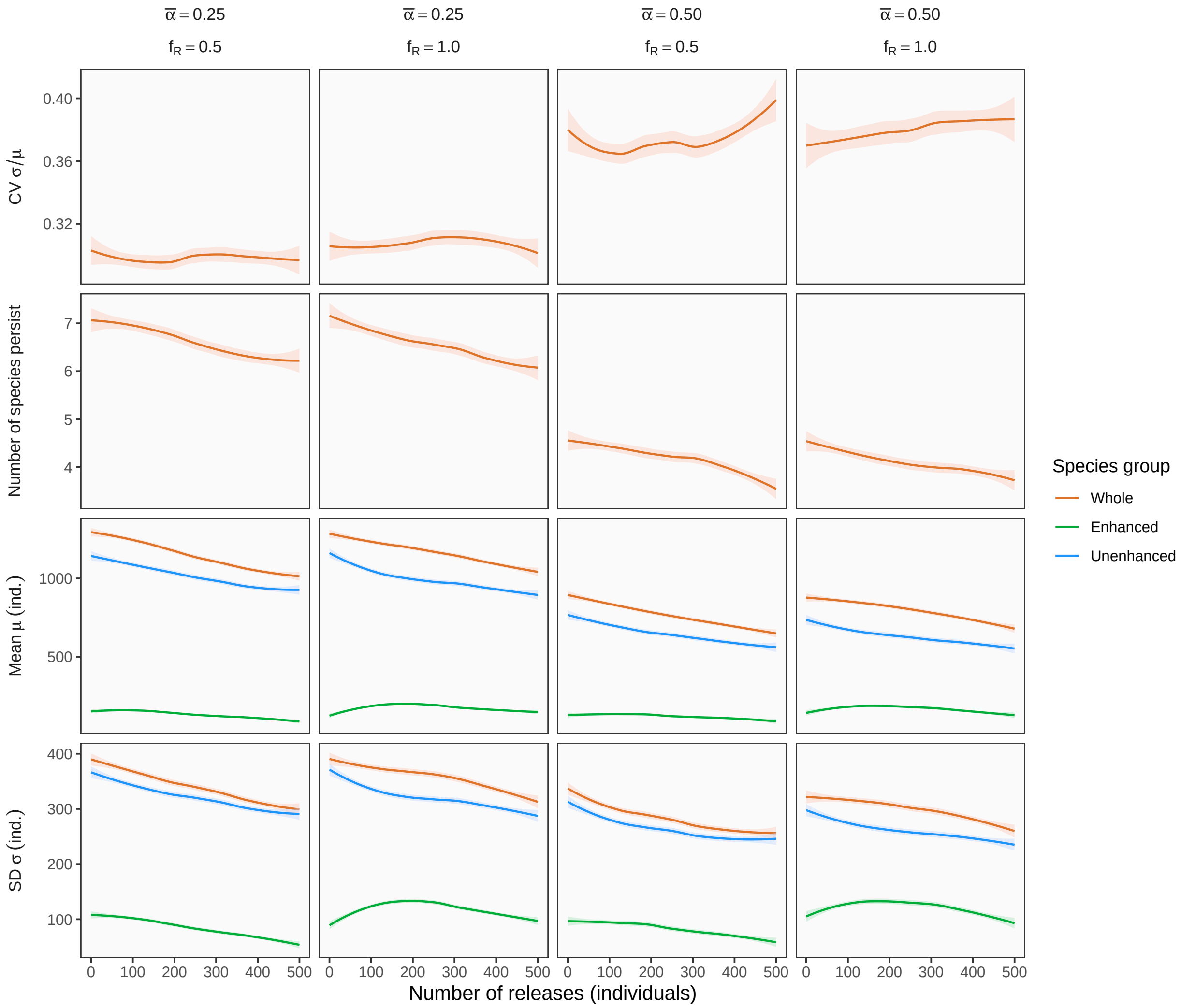

Figure S8: Theoretical predictions for the release effect in a ten-species community. Rows represent different response variables, and columns show distinct simulation scenarios with different strengths of interspecific competition ($\bar{\alpha}$) and relative fitness of captive-bred individuals (f_R). Lines are loess curves (mean) with shades indicating associated 95% confidence intervals. Other parameters are: number of species S = 10; intrinsic growth rate of an enhanced species r_1 = 1.5; maximum intrinsic growth rate of unenhanced species $r_{\max}$ = 2.5; environmental stochasticity σ_ε = 0.5; carrying capacity K = 400.

3.9 Theoretical prediction (r_1 = 2.5, K = 400)

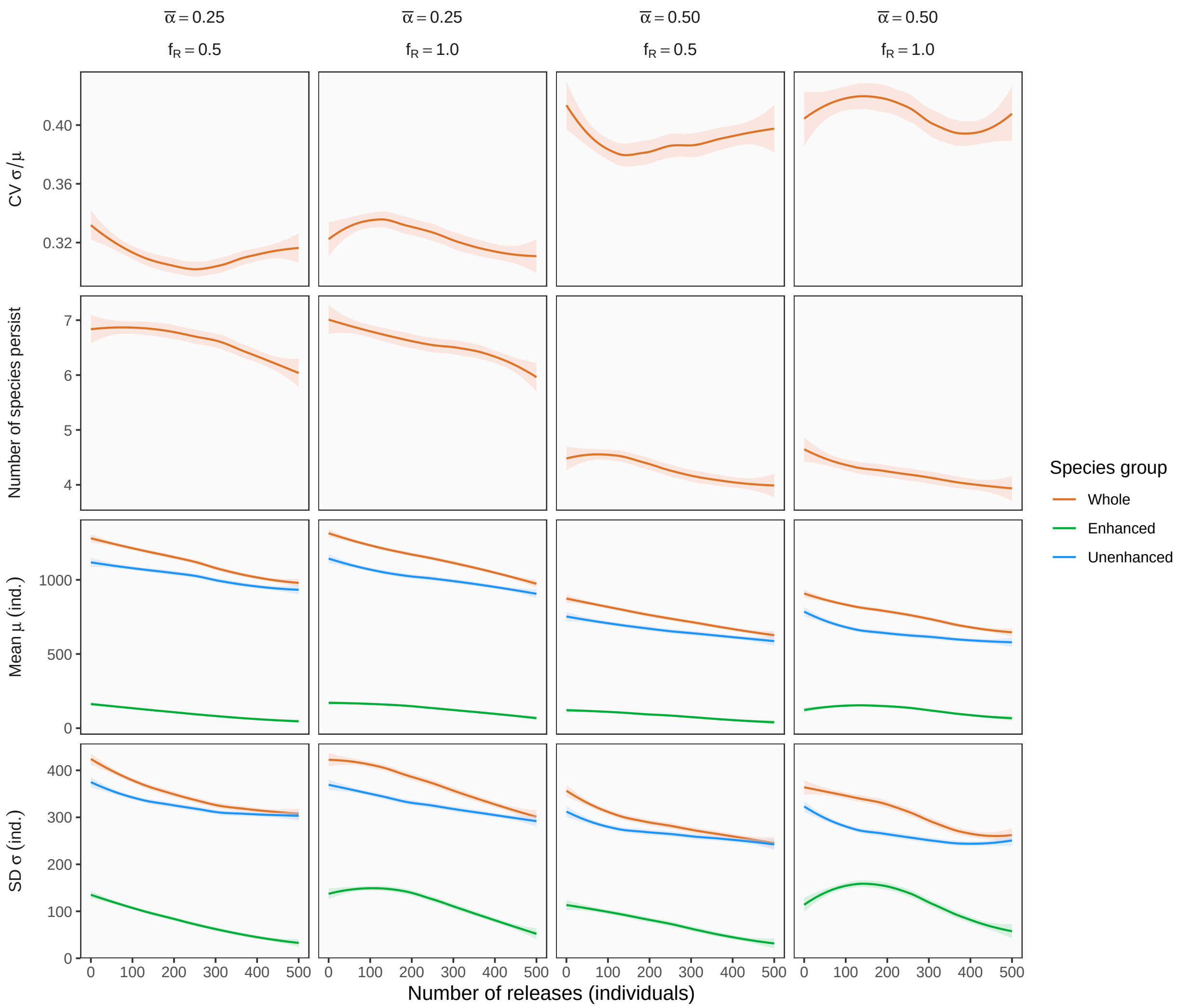

Figure S9: Theoretical predictions for the release effect in a ten-species community. Rows represent different response variables, and columns show distinct simulation scenarios with different strengths of interspecific competition ($\bar{\alpha}$) and relative fitness of captive-bred individuals (f_R). Lines are loess curves (mean) with shades indicating associated 95% confidence intervals. Other parameters are: number of species $S = 10$; intrinsic growth rate of an enhanced species $r_1 = 2.5$; maximum intrinsic growth rate of unenhanced species $r_{max} = 2.5$; environmental stochasticity $\sigma_\varepsilon = 0.5$; carrying capacity $K = 400$.

3.10 Theoretical prediction (r_1 = 3.5, K = 400)

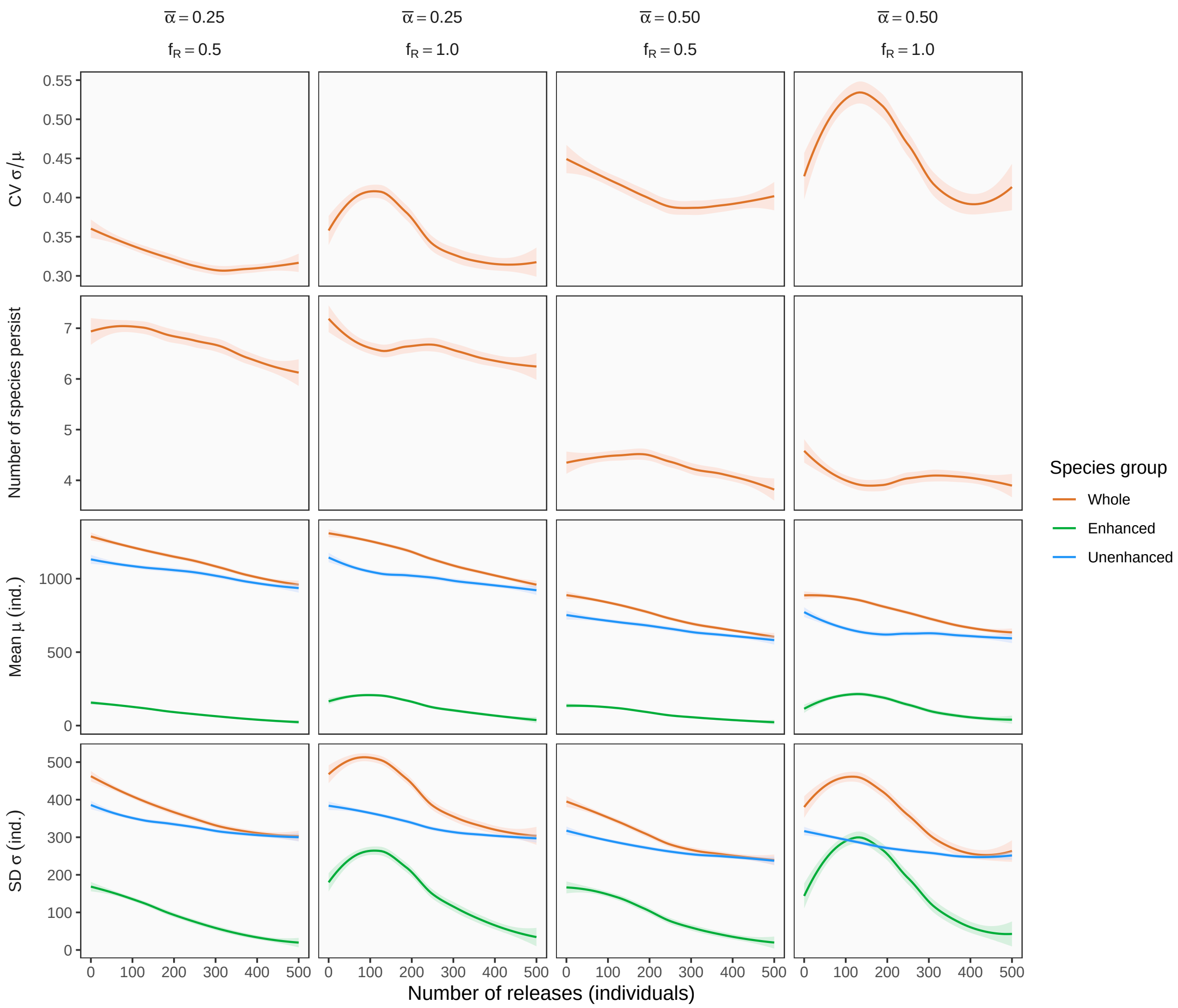

Figure S 10: Theoretical predictions for the release effect in a ten-species community. Rows represent different response variables, and columns show distinct simulation scenarios with different strengths of interspecific competition ($\bar{\alpha}$) and relative fitness of captive-bred individuals (f_R). Lines are loess curves (mean) with shades indicating associated 95% confidence intervals. Other parameters are: number of species S = 10; intrinsic growth rate of an enhanced species r_1 = 3.5; maximum intrinsic growth rate of unenhanced species r_{max} = 2.5; environmental stochasticity σ_ε = 0.5; carrying capacity K = 400.

3.11 Temporal dynamics of stream fish communities (whole community)

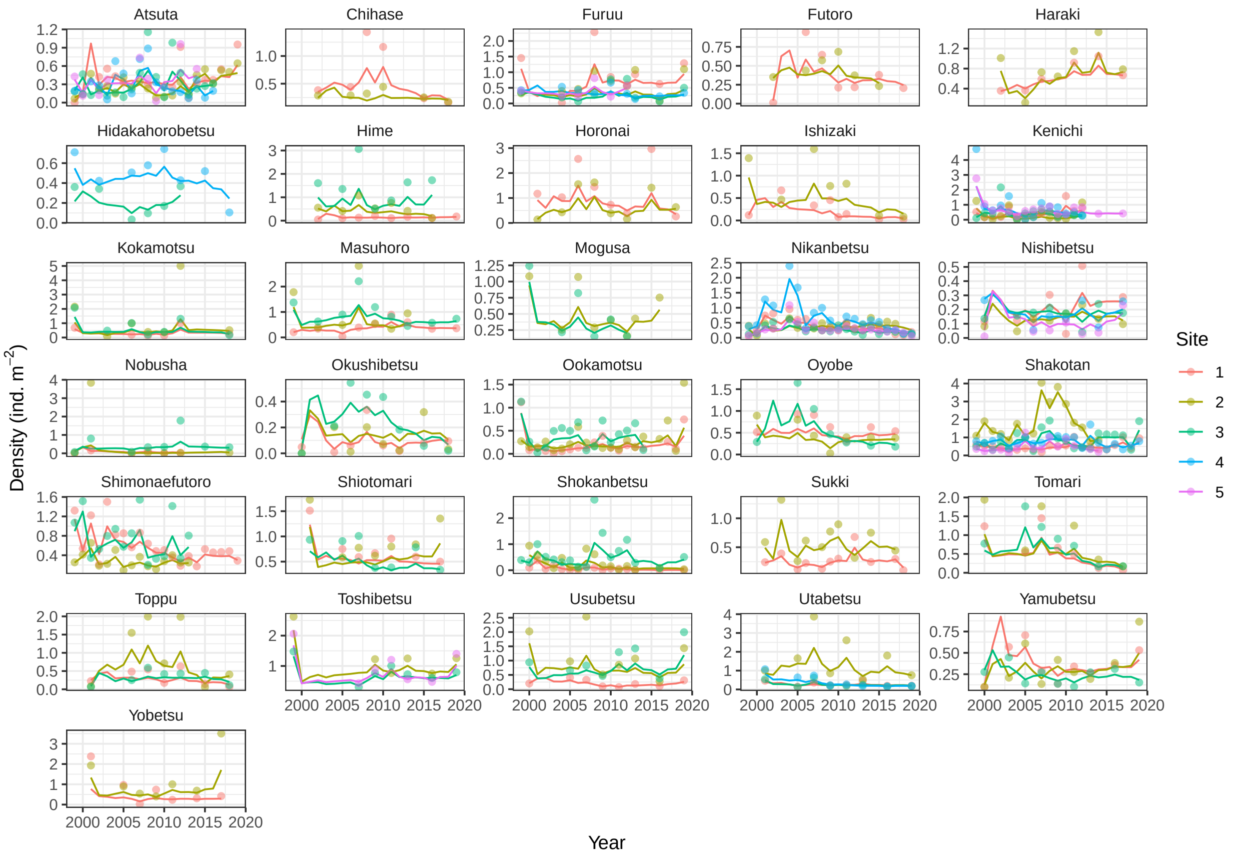

Figure S11: Temporal dynamics of stream fish communities (whole community) in Hokkaido, Japan. Dots represent observed density, and solid lines are the predicted values of the Bayesian state-space model. Panels correspond to individual watersheds, and colors distinguish sampling sites within a watershed.

3.12 Temporal dynamics of stream fish communities (masu salmon)

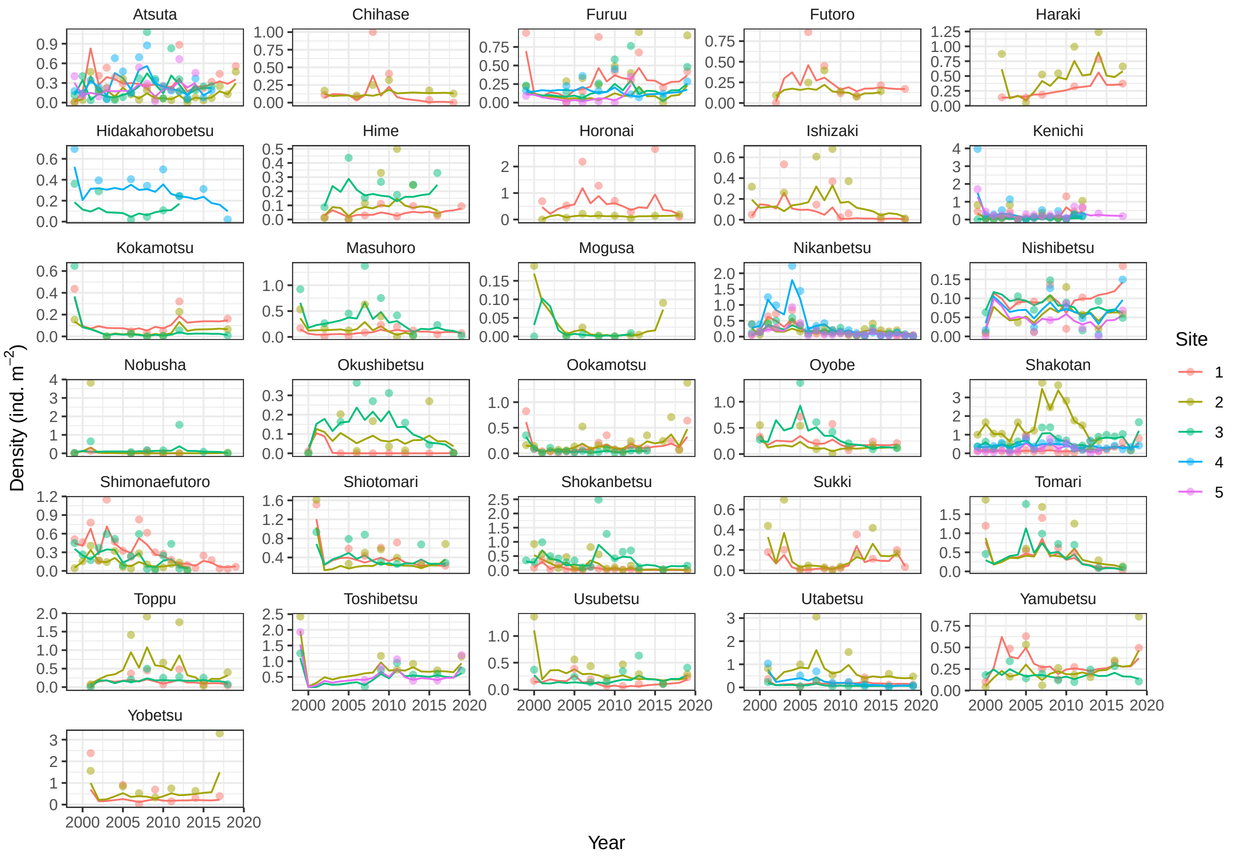

Figure S 12: Temporal dynamics of stream fish communities (masu salmon) in Hokkaido, Japan. Dots represent observed density, and solid lines are the predicted values of the Bayesian state-space model. Panels correspond to individual watersheds, and colors distinguish sampling sites within a watershed.

3.13 Temporal dynamics of stream fish communities (unenhanced species)

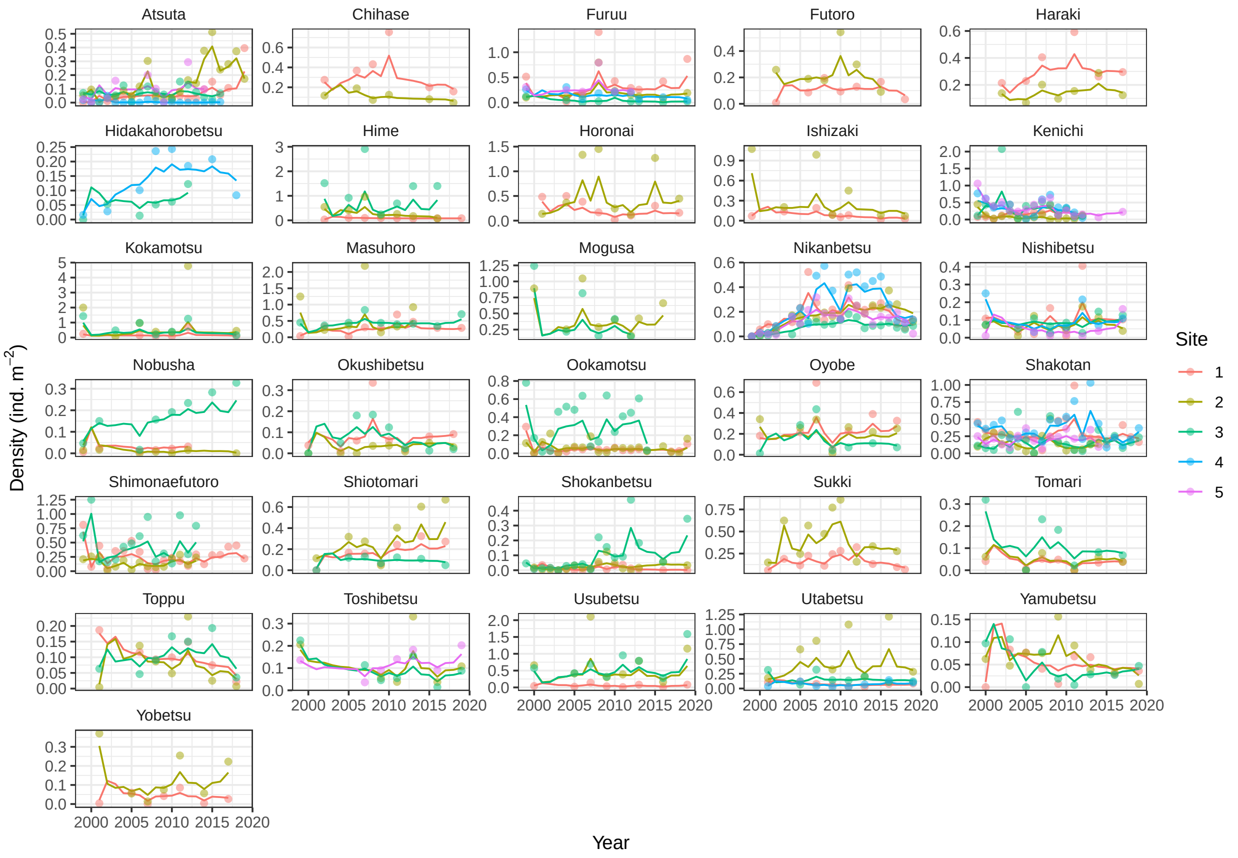

Figure S13: Temporal dynamics of stream fish communities (unenhanced species) in Hokkaido, Japan. Dots represent observed density, and solid lines are the predicted values of the Bayesian state-space model. Panels correspond to individual watersheds, and colors distinguish sampling sites within a watershed.

3.14 Environmental variables

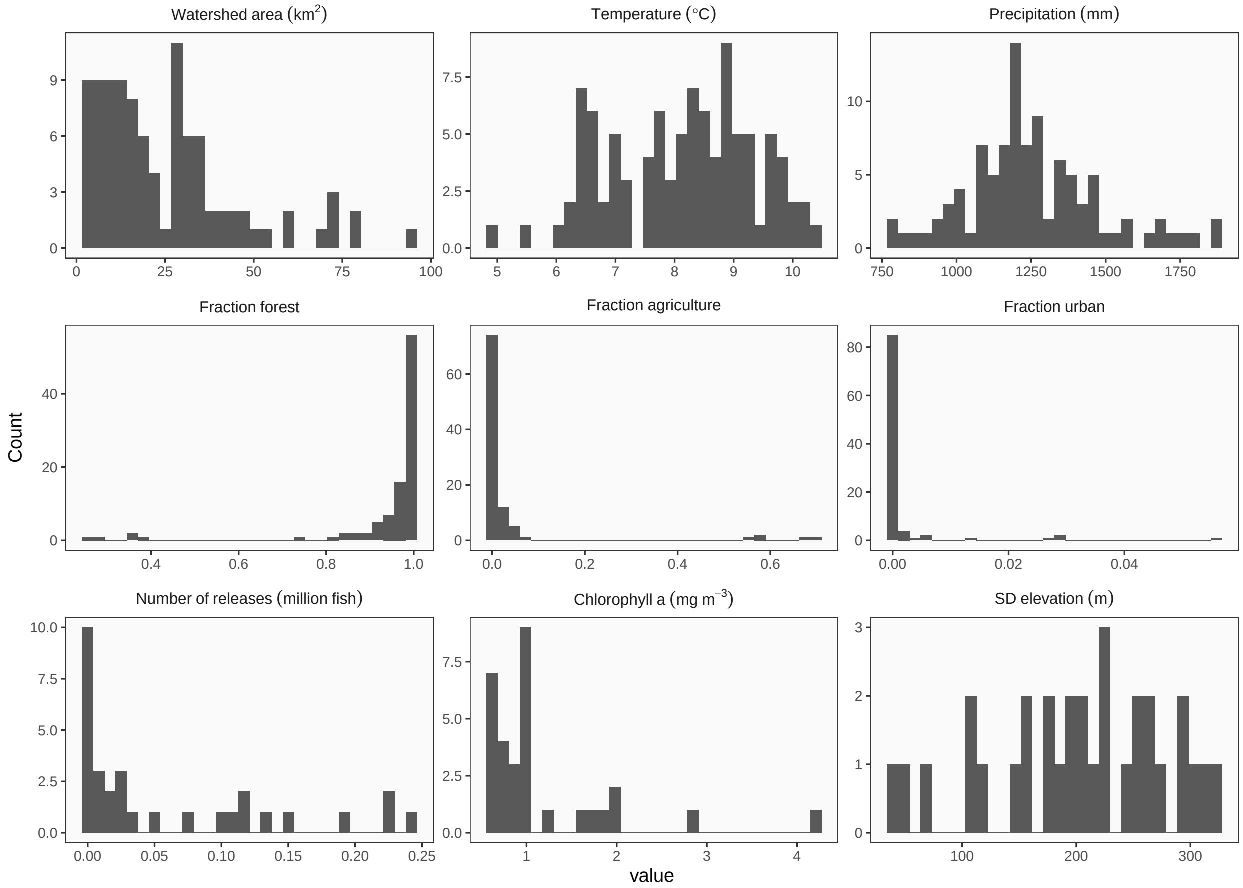

Figure S14: Distribution of environmental variables in protected watersheds. Note that the number of fish released, ocean productivity (chlorophyll a), and SD elevation are measured at the watershed level while others are measured at the site level.

3.15 Correlation plot

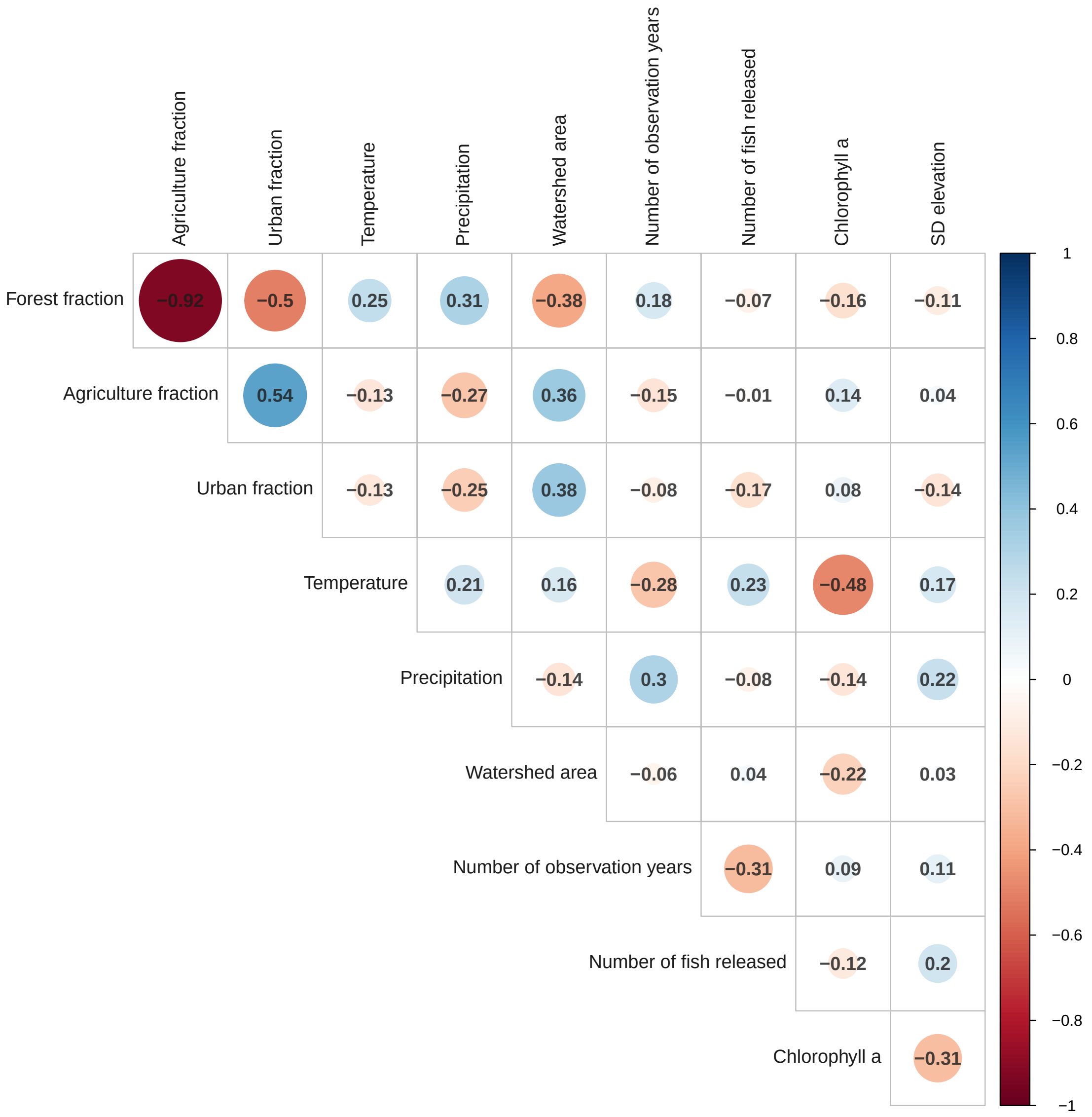

Figure S 15: Correlation plot among environmental variables in protected watersheds. Numbers indicate Spearman's rank correlations.

3.16 Co-occurrence matrix

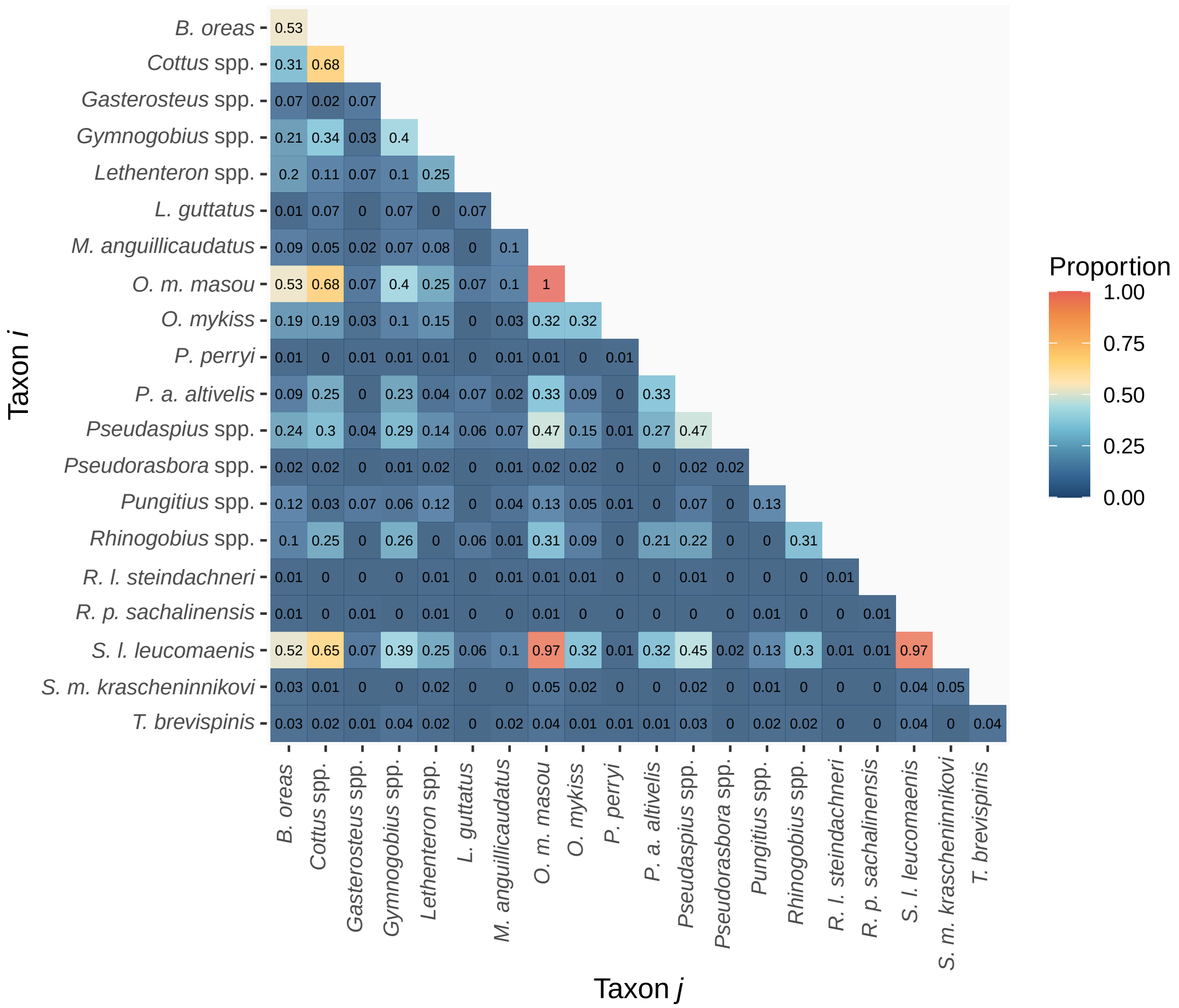

Figure S16: Co-occurrence matrix of stream fishes at 97 sites. Values in each cell indicate the proportion of co-occurrence for a given pair of species (off-diagonal) or the propotion of sites occurred for a given species (diagonal). Colors are proportional to the values.

3.17 Empirical estimates of competition coefficients

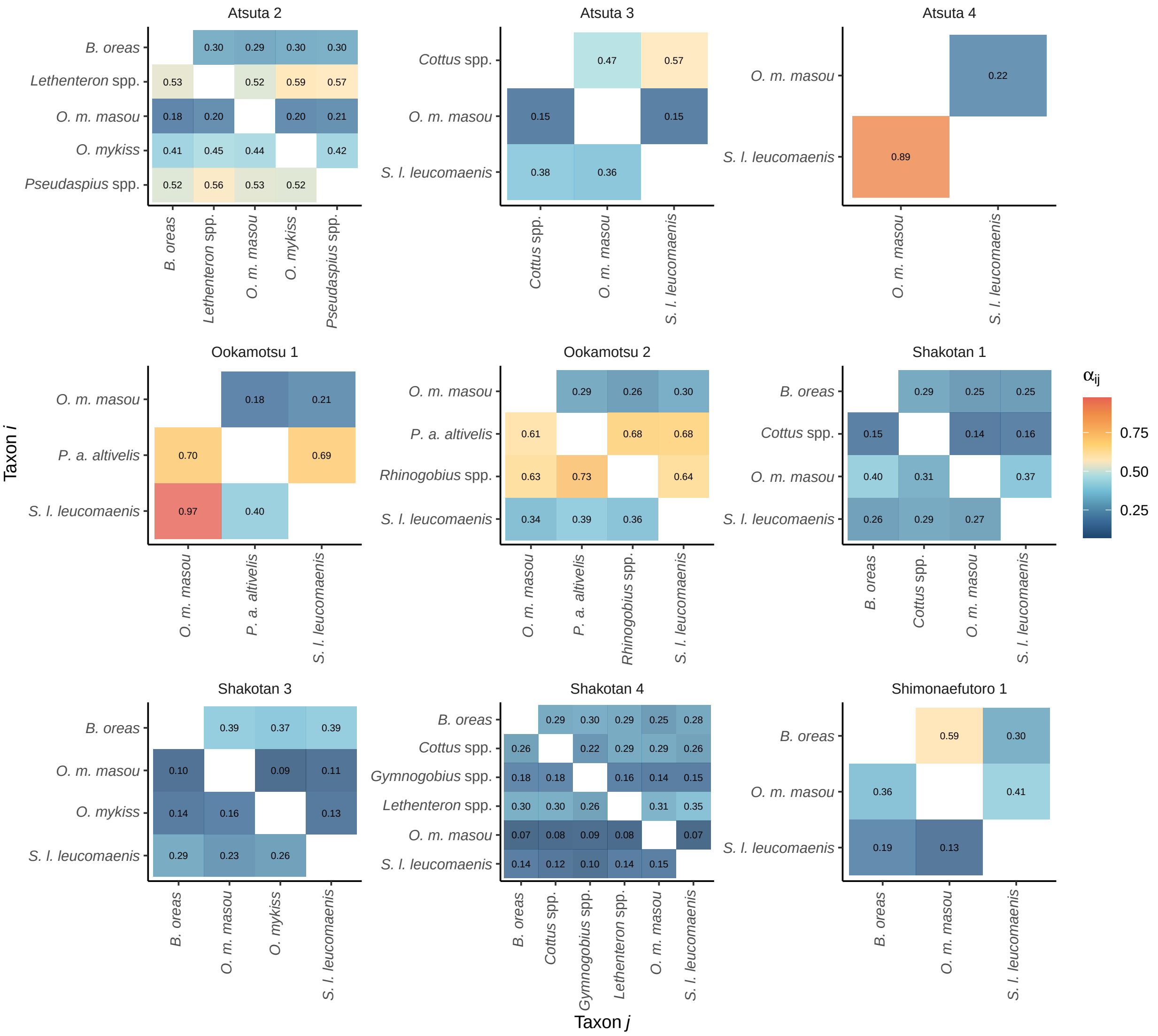

Figure S17: Estimates of competition coefficients α_{ij} in a multi-species Ricker model. Panels distinguish sites, and values in each cell indicate median estimates of α_{ij} for a given pair of species. Cells are colored in proportion to α_{ij}.

References

1. A. Goto, Ecological and morphological divergence of the freshwater sculpin *Cottus nozawae* Snyder-I. Spawning behavior and process of the devejopment in the post-hatching stage. *Bulletin of the Faculty of Fisheries Hokkaido University* **26**, 31–37 (1975).
2. A. Goto, Life history and distibution of a river sculpin, *Cottus hangiongensi*. *Bulletin of the Faculty of Fisheries Hokkaido University* **32**, 10–21 (1981).
3. Ministry of Environment of Japan, *Threatened wildlife of Japan, red data book 2014, brackish and fresh water fishes* (Ministry of Environment, Tokyo, 2014).
4. T. Natsumeda, T. Tsuruta, K. Iguchi, An evaluation of the ecological features of endangered freshwater fish commonly distributed in Japan. *Nippon Suisan Gakkaishi* **76**, 169–184 (2010).
5. FishBase, FishBase, https://www.fishbase.se/search.php, Accessed 2022/9/29.
6. D. Durante, A note on the multiplicative gamma process. *Statistics & Probability Letters* **122**, 198–204 (2017).
7. R. A. Myers, K. G. Bowen, N. J. Barrowman, Maximum reproductive rate of fish at low population sizes. *Canadian Journal of Fisheries and Aquatic Sciences* **56**, 2404–2419 (1999).
